# Protective effect of quercetin on cadmium-induced renal apoptosis through cyt-c/caspase-9/caspase-3 signaling pathway

**DOI:** 10.3389/fphar.2022.990993

**Published:** 2022-08-16

**Authors:** Ruxue Huang, Lulu Ding, Ying Ye, Ke Wang, Wenjing Yu, Bingzhao Yan, Zongping Liu, Jicang Wang

**Affiliations:** ^1^ College of Animal Science and Technology, Henan University of Science and Technology, Luoyang, China; ^2^ College of Veterinary Medicine, Yangzhou University, Yangzhou, China

**Keywords:** cadmium, kidney injury, apoptosis, quercetin, oxidative stress

## Abstract

Cadmium (Cd), a heavy metal, has harmful effects on animal and human health, and it can also obviously induce cell apoptosis. Quercetin (Que) is a flavonoid compound with antioxidant and other biological activities. To investigate the protective effect of Que on Cd-induced renal apoptosis in rats. 24 male SD rats were randomly divided into four groups. They were treated as follows: control group was administered orally with normal saline (10 ml/kg); Cd group was injected with 2 mg/kg CdCl_2_ intraperitoneally; Cd + Que group was injected with 2 mg/kg CdCl_2_ and intragastric administration of Que (100 mg/kg); Que group was administered orally with Que (100 mg/kg). The experimental results showed that the body weight of Cd-exposed rats significantly decreased and the kidney coefficient increased. In addition, Cd significantly increased the contents of Blood Urea Nitrogen, Creatinine and Uric acid. Cd also increased the glutathione and malondialdehyde contents in renal tissues. The pathological section showed that Cd can cause pathological damages such as narrow lumen and renal interstitial congestion. Cd-induced apoptosis of kidney, which could activate the mRNA and protein expression levels of Cyt-c, Caspase-9 and Caspase-3 were significantly increased. Conversely, Que significantly reduces kidney damage caused by Cd. Kidney pathological damage was alleviated by Que. Que inhibited Cd-induced apoptosis and decreased Cyt-c, Caspase-9 and Caspase-3 proteins and mRNA expression levels. To sum up, Cd can induce kidney injury and apoptosis of renal cells, while Que can reduce Cd-induced kidney damage by reducing oxidative stress and inhibiting apoptosis. These results provide a theoretical basis for the clinical application of Que in the prevention and treatment of cadmium poisoning.

## Introduction

Cadmium (Cd) is a silver-white heavy metal that is widespread in the environment. Cd pollution has the characteristics of a long half-life and long-lasting toxicity (Wang J. et al., 2020). Food and drinking water can cause the accumulation of Cd in the body. Cd has serious toxic effects on various tissues and organs of the body. For example, Cd has a toxic effect on the liver, kidney, bone, cardiovascular and reproductive system of mammals through bioaccumulation (Kazantzis, 2004; Varoni et al., 2017; Kim et al., 2018; Pérez Díaz et al., 2019; Zhu et al., 2019). The kidney is the primary organ in the body where Cd accumulates. In 1948, FRIBERG first reported the damage of Cd to the kidneys (Friberg, 1948). Cd exposure causes renal tubular reabsorption disorder (Gu et al., 2020). Cd leads to reactive oxygen species (ROS) to be accumulated in cells and damages the mitochondrial membrane potential (MMP) (Umar Ijaz et al., 2021). Cd exposure causes damage to mitochondria and ultimately induces apoptosis (Dong F. et al., 2021).

Quercetin (Que) is a flavonoid compound, found in a variety of plants, fruits, Chinese medicine and vegetables. A wide number of research have demonstrated that the most important biological function of Que is antioxidant. Que has a strong anti-tumor effect, which exerts anti-cancer functions through cell signal transduction pathways such as anti-oxidation, anti-proliferation and promotion of apoptosis (Zhou et al., 2016; Carrasco-Torres et al., 2017; Chen et al., 2018). The effects of carcinogens and mutagenic agents are inhibited, and the reproduction of malignant tumor cells is hindered. Que can eliminate DPPH, ·OH and ABTS+ *in vitro*, and inhibit lipid peroxidation in organs. Que can improve the body’s immunity and maintain the normal progress of various functions. Que has a protective effect on apoptosis (Feng et al., 2019).

Apoptosis is a self-destructive mechanism that exists in cells. However, excessive apoptosis can have detrimental effects on the body. Cd-induced apoptosis is mediated by mitochondria, apoptotic molecules will be released due to the increased permeability of the outer mitochondrial membrane, leading to apoptosis (Bauer and Murphy, 2020). Caspase-9 protein complex activates caspase-9, caspase-9 activates downstream caspase, such as caspase-3, which induces apoptosis (Choe et al., 2015). Previous studies have suggested that apoptosis pathways may be functionally involved in kidney injury. However, the specific signaling mechanism of apoptosis in Cd-induced nephrotoxicity is still unclear and needs further study.

In this work, we investigated the role of apoptosis in Cd-induced kidney damage in rats. Additionally, we addressed the protected effect of Que against Cd-induced kidney injury.

## Materials and methods

### Reagents

Uric acid (UA, C012-2-1), Creatinine (CRE, C011-2-1), Blood Urea Nitrogen (BUN, C013-2-1), Reduced glutathione (GSH, A006-2-1) and Malondialdehyde (MDA, A003-1-2) assay kits were from Jiancheng Bioengineering Institute (Nanjing, China). Anhydrous CdCl_2_ (purity: >99.95%) was from Aladdin Industrial Corporation. Que (purity: > 97%) was from RHAWN (Shanghai, China). RNA isolater Total RNA Extraction Reagent (R401-01), HiScript III RT SuperMix for qPCR (+gDNA wiper, R323-01), and ChamQ universal SYBR qPCR Master Mix (Q711-02) were from novozan Biotechnology (Nanjing, China). β-Actin and antibodies against Caspase-3, Caspase-9 and Cyt-c were from Servicebio (Wuhan, China). BeyoECL Star was from Beyotime Biotechnology (P0018AM, Shanghai, China). All other routine chemicals and solvents were of pure analytical grade.

### Animal experiments

Twenty-four 5-week-old male SD rats were adaptively reared for 1 week with adequate feed and drinking water. They were put into four groups at random, each with six rats weighing around 150 ± 5 g. A 12-h light/dark cycle was set. All animal experimental processes were approved by the Institute of Zoology and Medical Ethics Committee of Henan University of Science and Technology and they were strictly designed under the consideration of animal welfare (approval number HAUST 20015).

The experiment was carried out for 3 weeks. The body weight of rats was recorded every week, and they were treated as follows. The control group was given normal saline (10 ml/kg) every day. The Cd-treated group was injected with 2 mg/kg CdCl_2_ intraperitoneally. The Cd + Que group received 2 mg/kg CdCl_2_ by intraperitoneal injection and 100 mg/kg Que intragastrically. Que treatment group was administered orally with Que (100 mg/kg). The doses of Cd and Que were chosen based on previous studies (Yang S. H. et al., 2019; Liang et al., 2020). After 3 weeks, the rats were anaesthetized with ether and sacrificed. Kidneys were collected for further investigation.

### Weight and kidney coefficient determination

The rats’ body weight was measured every weekend. The effects of Cd and Que on the rat’s body weight were analyzed. The kidney of the rat was removed. The blood on the kidney surface was washed with saline. The excess water on the kidney was wiped clean, and the weight was weighed. The organ coefficient was calculated.

### Measure kidney function

Collected rat venous blood and left for a period of time, centrifuged 3000 r/min for 10 min, the serum was aspirated. The manufacturer’s guidelines were followed while measuring UA, CRE and BUN in rat serum using diagnostic kits. These results were measured spectrophotometrically.

### Measure antioxidant index

0.4 g kidney tissues were cut, grind on the ice at a ratio of kidney tissue (g): saline (ml) = 1:9, and centrifuged (3,000 rpm for 10 min) the supernatant was obtained. The contents of MDA and GSH in rat kidney tissues were assessed using diagnostic kits according to the manufacturer’s instructions. These results were measured spectrophotometrically.

### Histopathological studies

The fresh, morphological and structurally intact kidney tissues were fixed in 10% formalin for 48 h. Then rinsed with running water overnight and dehydrated with different concentrations of ethanol, transparent in xylene and embedded in paraffin. The paraffin blocks were cut with a microtome to a thickness of 5 μm. The kidney sections were stained with hematoxylin-eosin and mounted for microscope observation.

### TdT-UTP nick end labeling (TUNEL)

The prepared paraffin sections were washed twice with xylene and then dipped in graded ethanol. After washing with PBS, proteinase K solution was added to hydrolyze for 15 min at room temperature to remove tissue proteins. Wash with distilled water, add 0.1% triton dropwise to cover the tissue. Washed with PBS, incubated with buffer. TDT enzyme, dUTP, and buffer were mixed according to the instructions of the TUNEL kit, the tissue was then covered and incubated at 37 °C for 2 h. Drop by drop, DAPI staining solution was applied and incubated in the dark for 10 min. The sections were washed in PBS and mounted with anti-fluorescence quenching mounting media. A fluorescent microscope was used to examine the sections, and photographs were taken.

### Real-time PCR (RT-PCR)

Total RNA Extraction reagent was used to extract total RNA from rat kidney tissue and the total RNA concentration and purity were measured by a nucleic acid protein analyzer. The mRNA was reverse transcribed to synthesize cDNA. The primers were designed using Primer Premier 6 (Table 1). The instructions in the ChamQ universal SYBR qPCR Master Mix Kit for RT-PCR were followed. β-Actin was used as a control. The expression level of mRNA was analyzed using the 2^−ΔΔCT^.

**TABLE 1:** Primer sequences of real-time PCR target genes.

Target gene	Primers sequences (5′ → 3′)	Products	Genbank no.
*Caspase-9*	F:CTGAGCCAGATGCTGTCCCATA	106bp	NM_031632.2
	R:CCAAGGTCTCGATGTACCAGGAA		
*Caspase-3*	F:GCAGCAGCCTCAAATTGTTGACTA	156bp	NM_012922.2
	R:TGCTCCGGCTCAAACCATC		
*Cyt-c*	F:GGAGAGGATACCCTGATGGA	130bp	NM_012839.2
	R:GTCTGCCCTTTCTCCCTTCT		
*β-Actin*	F:AGGGAAATCGTGCGTGACAT	168bp	NM_031144.3
	R:CCTCGGGGCATCGGAA		

### Western blotting

Prepare suitable concentration of separating gel and 5% stacking gel. Electrophoresis after adding protein samples, and transferred onto PVDF membranes. Skim milk was used to seal the membranes for 2 h. The membranes were incubated with proportionally diluted caspase-9 (1:3000), caspase-3 (1:3000) and Cyt-c (1:2000) antibodies for 12 h at 4°C. As a loading control, *β*-*Actin* was used. After washing thrice with TBST, the membranes were incubated with secondary antibody for 2 h. The ECL chromogenic solution was uniformly put over the membrane after three TBST washes and incubated for 60 s. The membranes detected signals by the gel imaging equipment. Photos were taken for subsequent analysis.

### Statistical analysis

The result was expressed in the form of mean ± SEM. SPSS22.0 was used for statistical analysis. ANOVA was used to analyze the differences between multiple groups. LSD or Tamhane’s T2 method was used following the homogeneity test of variance. When *p* < 0.05 or *p* < 0.01, the difference is statistically significant.

## Result

### Effect of Que on Cd-induced changes in body weight and kidney coefficient

**FIGURE 1:**
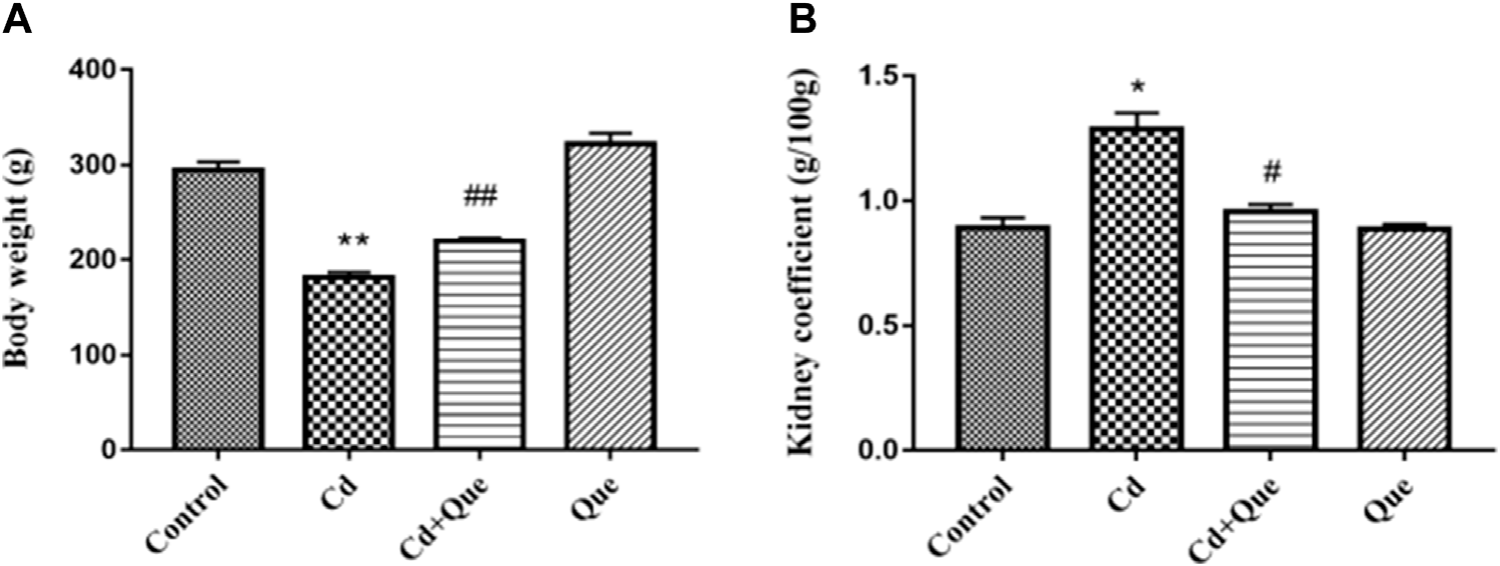
Effects of Quercetin (Que) on body weight and kidney coefficients in Cd-induced kidney injury rats. Changes in rat body weight **(A)** and kidney coefficients **(B)** were measured. Data were expressed as mean ± SEM. *n = 6*, *p < 0.05, **p < 0.01 indicates a significant or extremely significant difference compared to the control group; ^#^ *p* < 0.05, ^##^ *p* < 0.01 indicates a significant or extremely significant difference compared to the Cd group.

The body weight and kidney coefficient of rats in each group were compared. Results showed that Cd decreased the rat body weight and increased the renal coefficient. As presented in Figures 1A,B, compared with the control group, the body weight of the rats in the Cd group decreased by 61.5% (*p* < 0.01). The kidney coefficient in Cd-treated was 144% higher than that of the control (*p* < 0.05). Compared with the Cd group, the body weight of the rats in Cd + Que-treated group increased by 121% (*p* < 0.01) and the kidney coefficient decreased by 73.8% (*p* < 0.05).

### Cd causes kidney damage and Que reduces kidney damage

**FIGURE 2:**
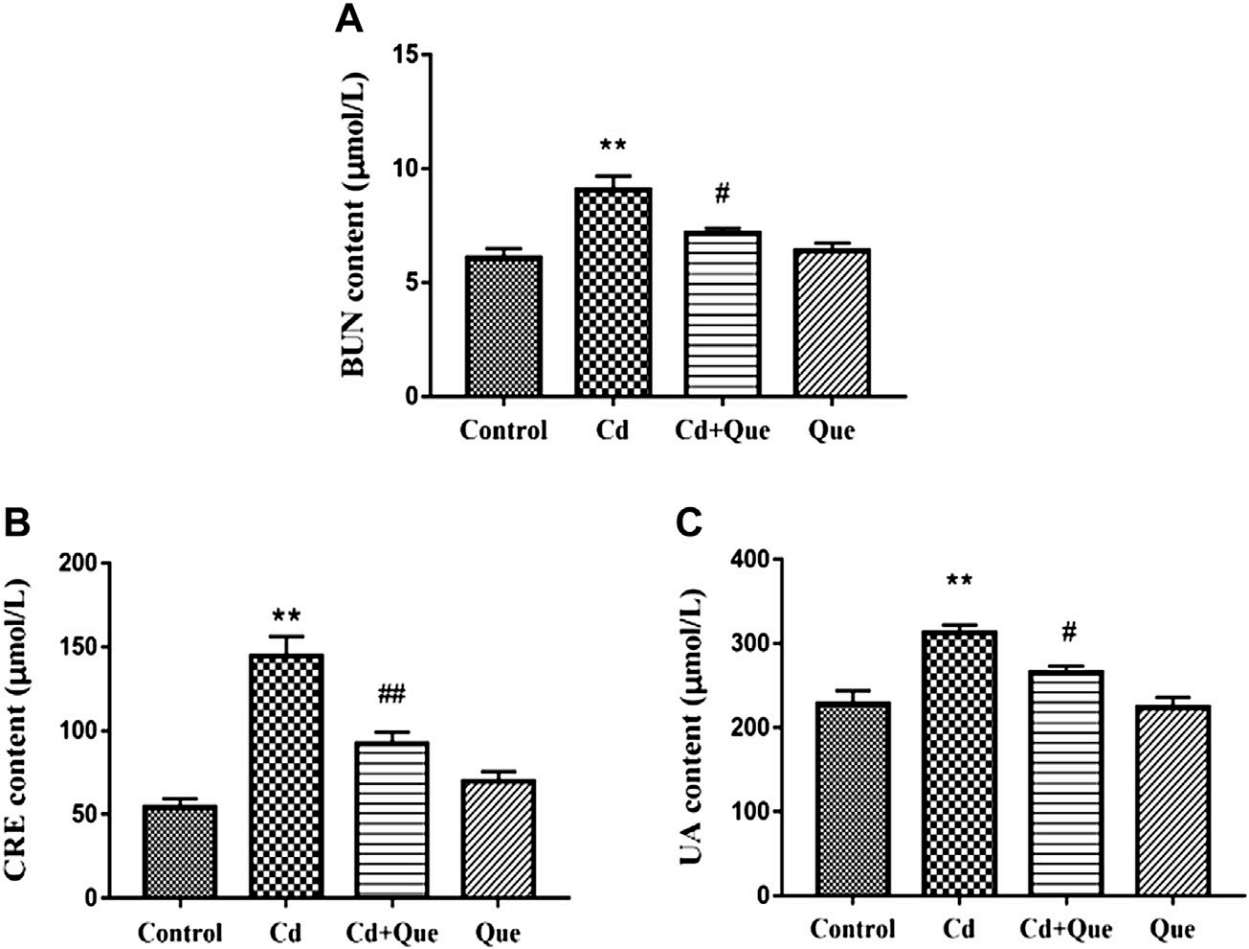
Effects of Quercetin (Que) on renal function in Cd-induced kidney injury rats. The content of BUN in serum **(A)**, the content of CRE in serum **(B)** and the content of UA in serum **(C)** were measured. Data were expressed as mean ± SEM. *n* = 6, *p = 0.05, **p = 0.01 indicates a significant or extremely significant difference compared to the control group; ^#^ *p* = 0.05, ^##^ *p* = 0.01 indicates a significant or extremely significant difference compared to the Cd group.

Renal function related indicators were measured. As shown in Figures 2A,C, compared with the control group, the contents of BUN, CRE and UA in the serum of the rats in the Cd group were significantly increased (*p* < 0.01). BUN, CRE and UA content increased by 149%, 265% and 137%, respectively. However, the contents of BUN and UA in the Cd + Que-treated were lower than those of the Cd-treated group by 78.9% and 84.7% (*p* < 0.05), Also, the CRE content decreased by 63.7% (*p* < 0.01). The difference between the control and Que groups was not significant.

### Effects of Cd and Que on the contents of GSH and MDA in rat kidney tissues

**FIGURE 3:**
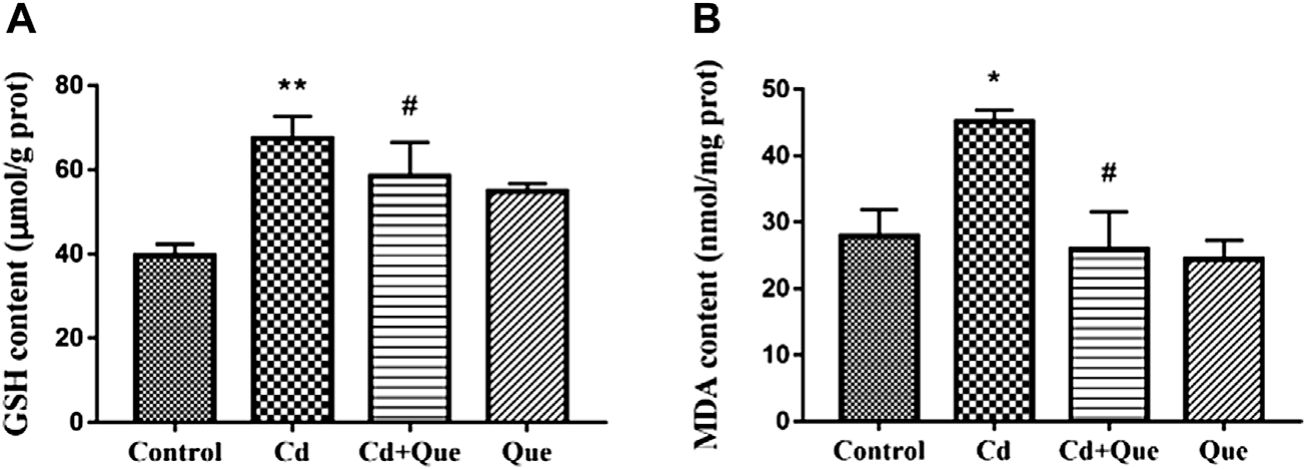
Effects of Quercetin (Que) on oxidative stress in Cd-induced kidney injury rats. The activity of GSH **(A)** and the content of MDA **(B)** in rats' kidney tissues were measured. Data were expressed as mean ± SEM. *n* = 6, *p < 0.05, **p < 0.01 indicates a significant or extremely significant difference compared to the control group; ^#^ *p* < 0.05, ^##^ *p* < 0.01 indicates a significant or extremely significant difference compared to the Cd group.

Cd induces oxidative damage to the kidney and lipid peroxidation. Que can weaken the kidney damage. As presented in Figures 3A,B, the contents of GSH and MDA in the renal tissues increased by 170% and 162% in Cd-treated group compared with the control group. The contents of GSH and MDA in the Cd + Que-treated group decreased by 86.9% and 57.4% compared with the Cd-treated group (*p* < 0.05).

### Observation of renal tissue pathological section

**FIGURE 4:**
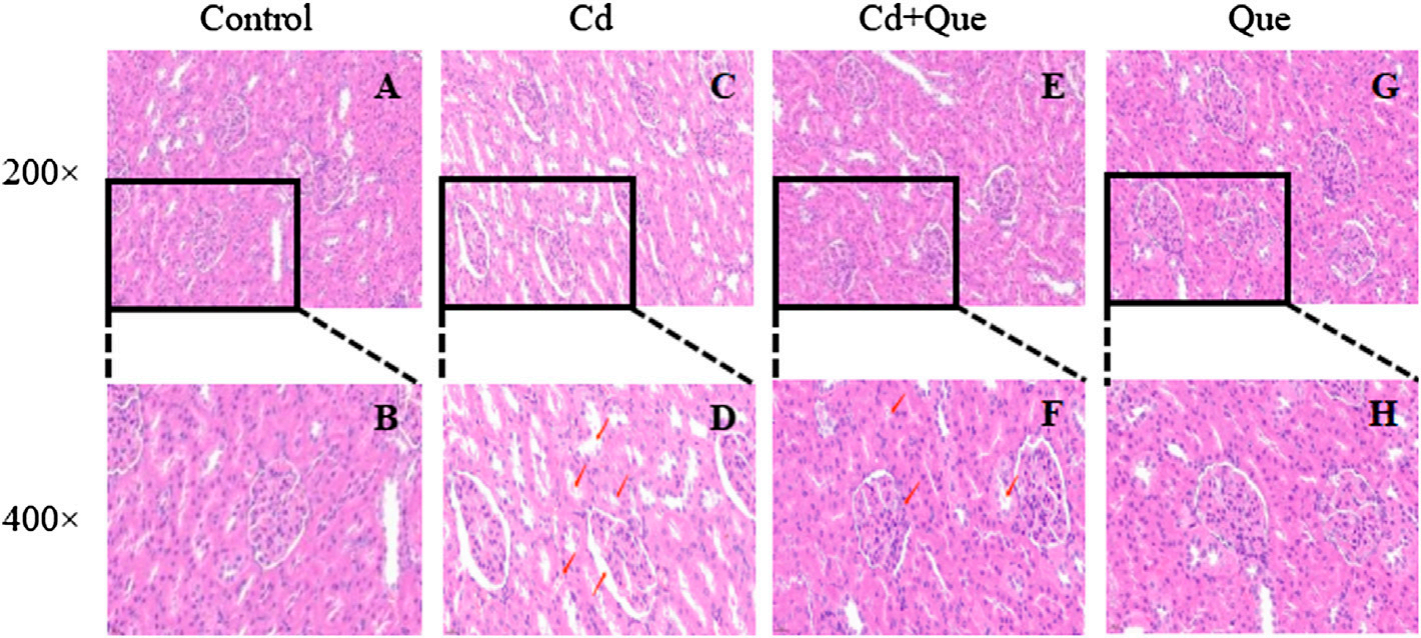
Effects of Quercetin (Que) on histopathological changes in kidney tissues in Cd-induced kidney injury rats. Representative H&E sections of the kidney. **(A)** control 200× **(B)** control 400× **(C)** Cd, 2 mg/kg b.w. 200× **(D)** Cd, 2 mg/kg b.w. 400× **(E)** Cd, 2 mg/kg b.w. + Que, 100 mg/kg, 200× **(F)** Cd, 2 mg/kg b.w. + Que, 100 mg/kg, 400× **(G)** Que, 100 mg/kg, 200× **(H)** Que, 100 mg/kg, 400×.

The size and shape of the glomeruli in the control group were normal with clear borders (Figures 4A,B). In the Cd group (Figures 4C,D), the renal tubular lumen was blocked, the renal tubules were dilated, the renal tubular epithelial cells were necrotic and fell into the lumen, the renal interstitium was congested, and the glomerular cyst cavity became obvious pathological changes. In the Cd and Que co-treatment group (Figure 4E and F), the size and shape of the glomerulus return to normal, some of the cysts became smaller and adhered to the glomerulus, the tubular lumen was narrowed, and some tubular epithelial cells swelled and fell off into the lumen. The degree of damage was relieved compared with the Cd group. In the Que group (Figure 4G and H), the morphology and structure of the glomeruli were normal, with clear borders and no pathological changes.

### Effects of Cd and Que on apoptosis of rat kidney cells

**FIGURE 5:**
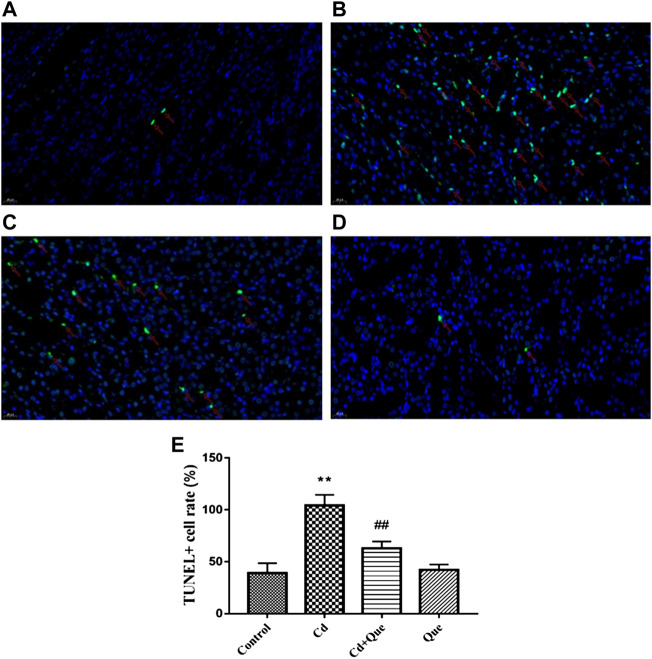
Effects of Quercetin (Que) on the rate of TUNEL-positive apoptosis in Cd-induced kidney injury rats. **(A)** TUNEL-positive cells in the control group 400×. **(B)** TUNEL-positive cells in the Cd-treated group 400×. **(C)** TUNEL-positive cells in the Cd + Que-treated group 400×. **(D)** TUNEL-positive cells in the Que-treated group 400×. The green particles are apoptotic cells, and the blue particles are normal or proliferating cells. **(E)** The percentage of TUNEL-positive cells in control and Cd groups within or without Que using TUNEL staining. Data were expressed as mean ± SEM. *n* = 3, **p < 0.01 indicates extremely significant difference compared to the control group; ^##^ *p* < 0.01 indicates extremely significant difference compared to the Cd group.

The number of TUNEL apoptotic cells in Cd-treated kidney tissue (Figure 5B) was considerably higher than in the control group (Figure 5A), an increased of 267%. The ratio of TUNEL apoptotic cells in kidney tissue co-treated with Que and Cd (Figure 5C) was lower than that in Cd-treated kidney cells, an decreased 60.4%. The number of TUNEL apoptotic cells in renal tissue did not differ significantly between the control and Que groups (Figure 5A and D).

### Effects of Cd and Que on mRNA expression of apoptosis-related gene

**FIGURE 6:**
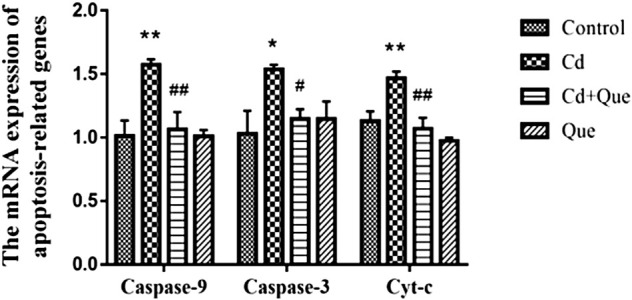
Effects of Quercetin (Que) on mRNA expression of apoptosis-related genes in Cd-induced kidney injury rats. The mRNA expression of Caspase-9, Caspase-3 and Cyt-c in kidney tissue was reduced by Que. Data were expressed as mean ± SEM. *n* = 3, *p < 0.05, **p < 0.01 indicates a significant or extremely significant difference compared to the control group; ^#^ *p* < 0.05, ^##^ *p* < 0.01 indicates a significant or extremely significant difference compared to the Cd group.

The RT-PCR results showed that Cd exposure led to 130%, 155% and 149% increases in Cyt-c, Caspase-9 and Caspase-3 mRNA expression in kidney tissue, respectively. However, the intervention of Que significantly (*p* < 0.05) or extremely significantly (*p* < 0.01) decreased Cd-induced Cyt-c, Caspase-9 and Caspase-3 mRNA expression levels (Figure 6) by 73%, 67.6% and 74.6%.

### Cd induces apoptosis of kidney tissues and Que inhibits Cd-triggered apoptosis

**FIGURE 7:**
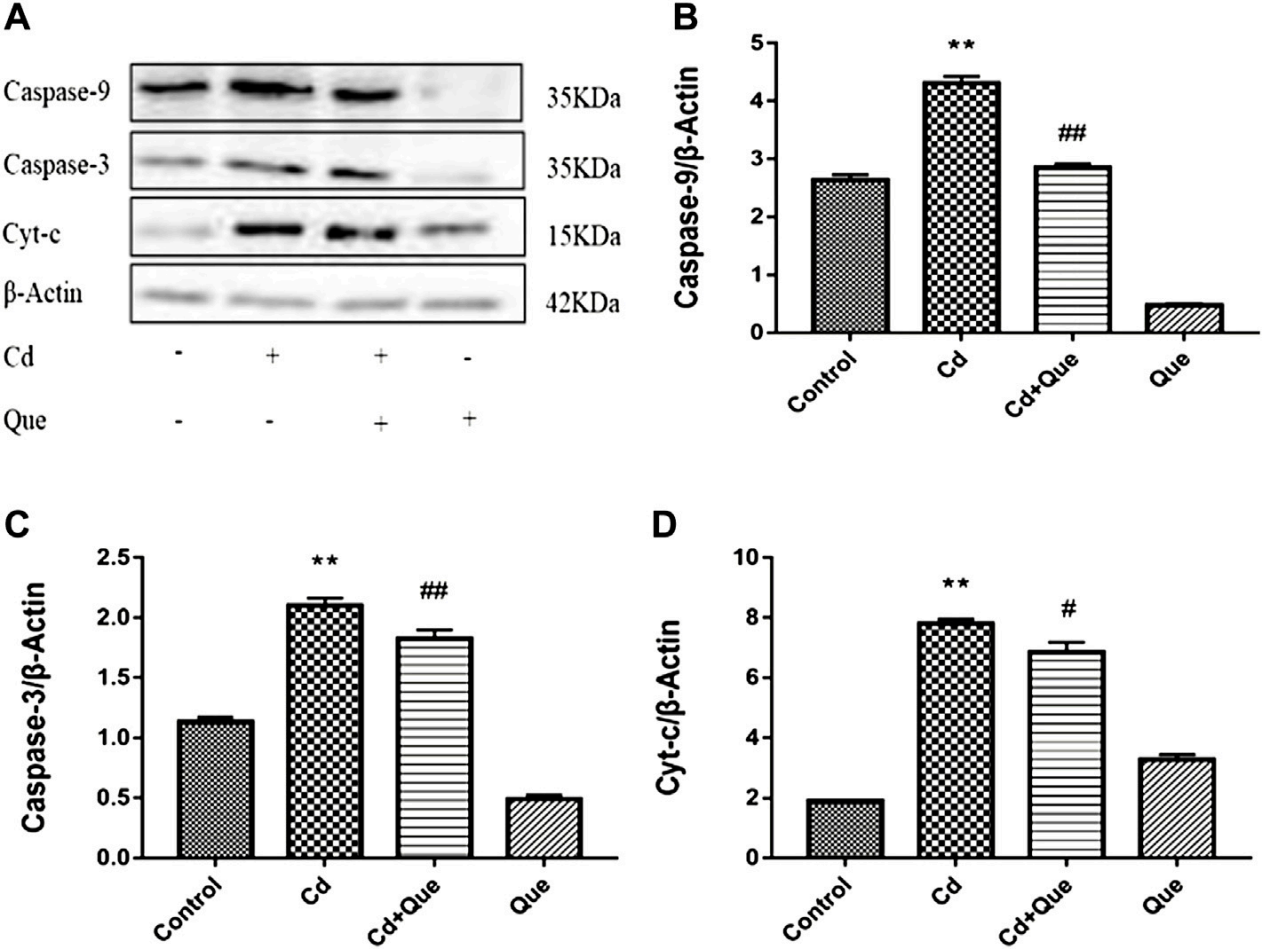
Effects of Quercetin (Que) on the levels of apoptosis-related proteins in Cd-induced kidney injury rats. **(A)** Western blotting was used to determine the amounts of Caspase-9, Caspase-3, and Cyt-c proteins, with β-Actin as a control. Quantitative analysis for Caspase-9 **(B)**, Caspase-3 **(C)** and Cyt-c **(D)**. The results reflect the mean ± SEM of three separate experiments. **p < 0.01 indicates extremely significant difference compared to the control group; ^#^ *p* < 0.05, ^##^ *p* < 0.01 indicates a significant or extremely significant difference compared to the Cd group.

The research showed that Cd exposure obviously enhanced the level of Cyt-c, Caspase-9 and -3 proteins in rats’ kidney tissue (Figure 7). Compared with the control group, the increase was 413%, 162% and 185%. Compared with Cd-treated group, the expression levels of Caspase-9 and -3 proteins reduced by 66.3 and 87% in Cd + Que-treated group (*p* < 0.01). The protein expression of Cyt-c decreased by 88% (*p* < 0.05).

## Discussion

In this study, we explored the role of apoptosis in kidney injury induced by Cd and the protective effect of quercetin. The damage mechanism of Cd to tissues is very complicated. Numerous studies have shown that Cd leads to kidney cell apoptosis, which mainly occurs through mitochondria-mediated signaling pathways (Indran et al., 2011). However, the detailed apoptotic signal of Cd-induced apoptosis in rat kidney cells is still unclear. Que has biological functions, such as anti-cancer, anti-inflammatory and anti-oxidant (Li et al., 2016), but the protective mechanism against Cd nephrotoxicity is still unclear.

Body weight reflects animal’s health status and absorption of nutrients, the decline in the rat’s health status is reflected by weight loss (Bhattacharya and Haldar, 2012). Consistent with the results of previous studies (Wang et al., 2021), the changes in rat body weight in this experiment showed that the heavy metal Cd hindered the growth of rats, while Que alleviates the toxicity of Cd in rats. The growth inhibition of rats may be due to the insufficient digestion and absorption of nutrients in intestinal mucosa caused by Cd (Eriyamremu et al., 2005). The kidney coefficient is used as a necessary test item for toxicology research. Increased organ coefficients indicate organ congestion and edema, and decreased organ coefficients indicate organ atrophy and other degenerative changes. Studies have shown that long-term exposure to 10 mg/kg of Cd increased liver and spleen weight (Tu et al., 2007). Here, we observed that kidney coefficients were increased by Cd, and Que antagonized the increase in kidney weight caused by Cd.

UA is the metabolic end product formed by the decomposition of nucleic acid into purine and then oxidized in the liver. Hypertension, obesity and renal disease can manifest as elevated UA (So and Thorens, 2010). UA can cause hippocampal inflammation, leading to cognitive dysfunction (Shao et al., 2016). CRE is the metabolite of muscle. Elevated serum CRE was found only in severely damaged kidneys (Stark, 1980). The end product of protein metabolism is BUN. Once the kidney is damaged, the glomerular filtration rate will decrease, resulting in increased BUN levels (Lee et al., 2006). Elevated serum levels of UA, CRE and BUN were associated with Cd-induced kidney damage. The amount they excrete is closely related to glomerular filtration and renal tubular reabsorption (Macedo, 2011). Luo et al. found that Cd exposure raised the serum CRE and BUN at 1 mg/kg, suggesting kidney filtration failure (Luo et al., 2016). The study by Iserhienrhien et al. also confirmed the damage of Cd exposure on rat renal function (Iserhienrhien and Okolie, 2022). Consistent with the research results of the above scholars, Cd increases the content of kidney function-related indicators, leading to renal function damage. The index of the co-treatment group decreased, which proved that Que can reduce the renal function damage caused by Cd.

Cd causes increased levels of free radicals in cells, which ultimately cause tissue damage (Skipper et al., 2016). Cd induces a large number of reactive oxygen species (ROS) in mitochondria by inhibiting cellular respiration (Wang et al., 2004). ROS causes cellular oxidative damage (Zhu et al., 2016; Vodošek Hojs et al., 2020). GSH has the capability to remove heavy metal Cd or ROS. Loss of reducibility by GSH after binding to Cd cannot scavenge ROS, which accumulates in large amounts *in vivo* and leads to oxidative damage (Hart et al., 2001). Some studies have shown that Cd pollution increased GSH levels (Maity et al., 2018; Dong A. et al., 2021). GSH levels in Cd-treated rats were greater than in control rats in the current study. However, some reports indicated that Cd exposure significantly reduced GSH levels, which is inconsistent with some research findings (Jihen el et al., 2010; Renugadevi and Prabu, 2010; Owumi et al., 2019). The reason may be that after Cd enters the body, in order to resist the oxidative damage caused by stress, a large amount of GSH is produced in the body through chelation to remove free Cd^2+^. In addition, the difference may also be related to factors such as dosage, duration of exposure, and age of animals. Oxidative stress causes lipid peroxidation in animal and plant cells. MDA is a natural product of lipid oxidation and is considered to be one of the biomarkers of oxidative stress (Zhang et al., 2014). Zhang et al. demonstrated that Cd increased MDA content by disrupting the antioxidant system of shrimp (Zhang et al., 2021). The results of Messaoudi et al. showed that the MDA concentration increased in rat kidney tissue of Cd-treated (Messaoudi et al., 2009). The current investigation found that the content of MDA in the kidney tissue of the Cd group was higher than that of the control group. Further results showed that Cd promoted lipid oxidation in kidney cells. This is consistent with the previous findings of our group (Wang et al., 2014). However, studies have shown that Que can alleviate cadmium-induced kidney damage by reducing cell membrane lipid peroxidation and enhancing total antioxidant capacity (Yuan et al., 2016). In the HepG2 cell model, Que eliminated lipid droplets and reduced total cholesterol and triglyceride levels, suggesting that Que can restore NAFLD by reducing oxidative stress and improving lipid metabolism (Yang H. et al., 2019). GSH and MDA levels in the Cd + Que-treated group were significantly lower than in the Cd-treated group. We found that Que reduced the oxidative damage and intracellular lipid oxidation induced by Cd, and exerted a protective effect on rat kidney tissue.

Histopathological changes could directly reflect the damage to kidney tissue. Cd caused significant histomorphological changes in the kidney, a similar phenomenon was also observed by Owumi et al. (Owumi et al., 2019). It is speculated that it may be related to the excessive accumulation of free radicals in the body caused by Cd exposure (Jihen el et al., 2009). Cd could regulate the body’s enzymatic activity to induce the accumulation of ROS (Zhong et al., 2015), leading to oxidative damage (Zhu et al., 2020), which in turn leads to pathological changes in the morphological structure of kidney tissue. Que is an effective scavenger of ROS and RNS. It has been reported that Que can scavenge ROS directly *in vitro* when the concentration of Que is in the range of 5-50 μM (Saw et al., 2014). In addition, Que can also downregulate ROS-induced oxidative stress by modulating signaling pathways (Veith et al., 2017). In this study, pathological sections showed that Cd caused severe damage to renal tissue, such as Enlarged glomerular cavity, tubular lumen obstruction and renal interstitial congestion. Nonetheless, Que attenuated Cd-induced renal lesions.

Cd causes apoptosis *in vivo* and *in vitro*, according to numerous studies (El-Baz et al., 2015; Dai et al., 2018; Wang C. et al., 2020). Consistent with previous studies, we also proved that Cd triggered caspase-dependent apoptosis in kidney cells. However, whether Que could inhibit Cd-induced apoptosis in renal tissue remains unclear. Mitochondria release cytochrome c (Cyt-c) via increased Bax levels (Song et al., 2016). Cyt-c is a sign of apoptosis (Zhang et al., 2017). And caspase-9 is activated. The current study found that activation of Caspase-9, an important indicator of apoptosis induced by the mitochondrial pathway, contributed to Cd-induced apoptosis in kidney cells (Choe et al., 2015). Caspase-9 is the initiator of the protease cascade. Activation of Caspase-3 is an important sign of apoptosis and Caspase-3 acts as the executor to induce apoptosis (Wasilewski and Scorrano, 2009). TUNEL-positive cells were found in larger numbers in the Cd-treated kidney than in the controls in this investigation. The rate of TUNEL-positive cells was significantly reduced under the intervention of Que. Cd exposure significantly increased the mRNA expressions of Cyt-c, Caspase-9 and Caspase-3 in kidney tissue, while those in the Que-treated group were significantly decreased compared with those in the Cd group. Consistent with the above results, Cd exposure obviously enhanced the level of Cyt-c, Caspase-9 and -3 proteins in rats’ kidney tissue. However, Cd-induced apoptosis was inhibited by Que. Overall, Cd triggers apoptosis in kidney cells, at least through the caspase-9-dependent mitochondrial signaling pathway. Que interferes with renal cell apoptosis induced by Cd and has a protective effect on rats’ kidney.

In summary, Cd decreases the body weight of rats, increases the renal coefficient, damages kidney function, causes oxidative stress, promotes cell lipid oxidation, and ultimately leads to apoptosis in kidney cells. Reversely, Que alleviated these changes, which has a protective effect against Cd-induced damage on rats’ kidney and inhibited apoptosis. To offer a theoretical foundation for the use of quercetin in the treatment of cadmium poisoning.

## Data Availability

The original contributions presented in the study are included in the article/Supplementary Material, further inquiries can be directed to the corresponding author.
