# Supplementary figures and images for: Protective effect of quercetin on cadmium-induced renal apoptosis through cyt-c/caspase-9/caspase-3 signaling pathway
Source: Front Pharmacol. 2022 Aug 16;13:990993. doi: 10.3389/fphar.2022.990993 (PMC9425064; doi:10.3389/fphar.2022.990993)

Control 200×


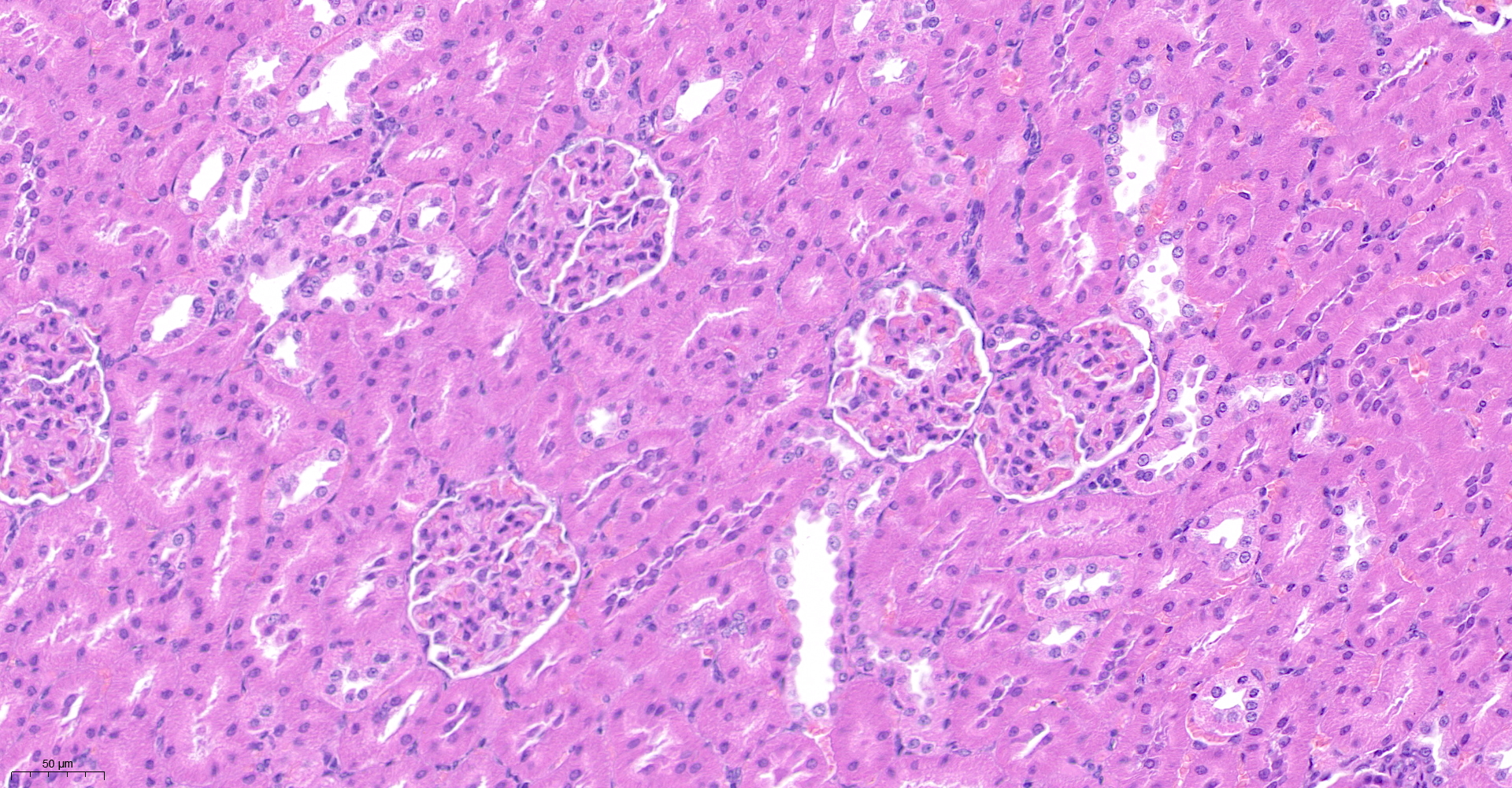


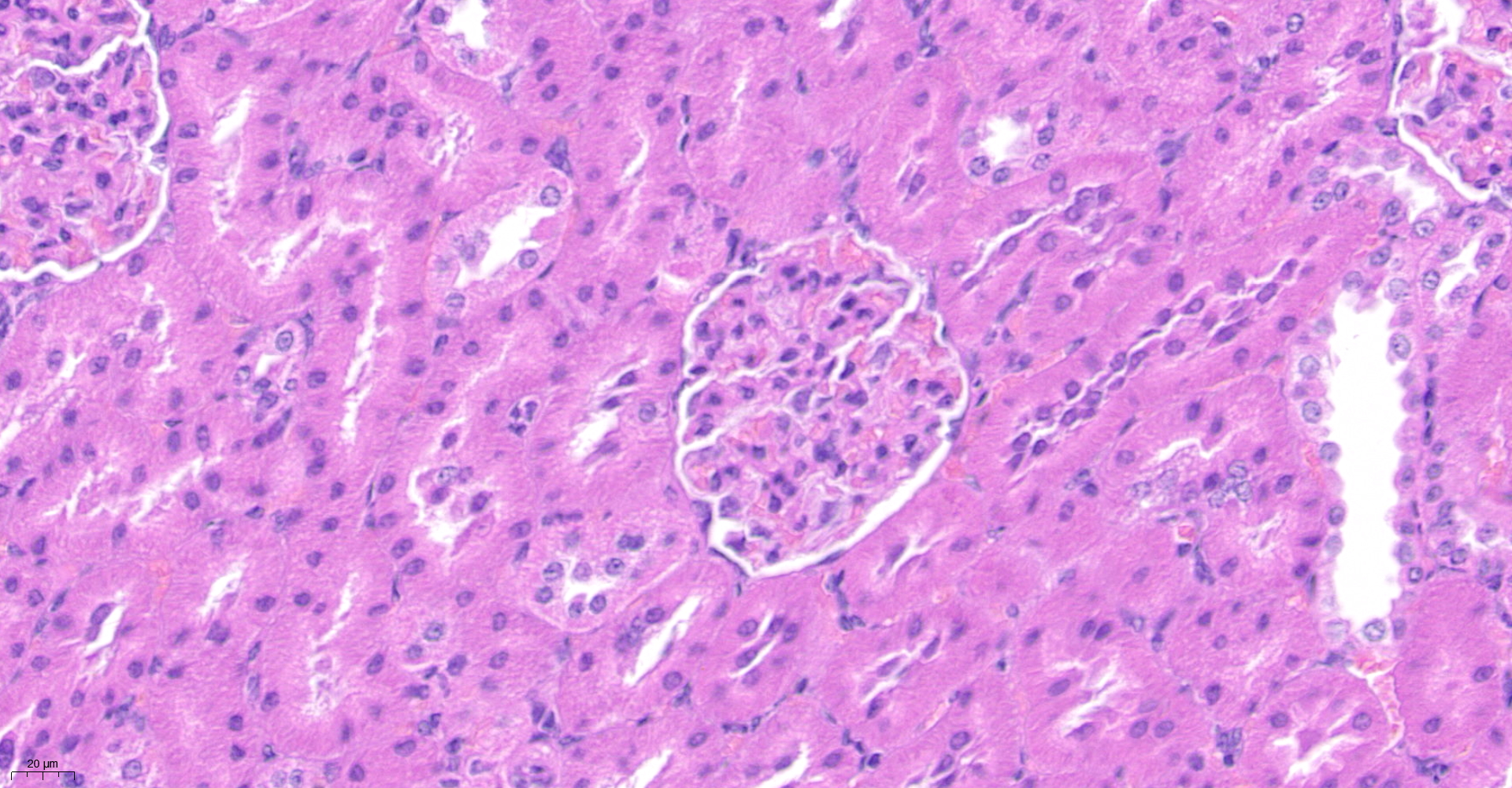
Control 400×

Cd 200×


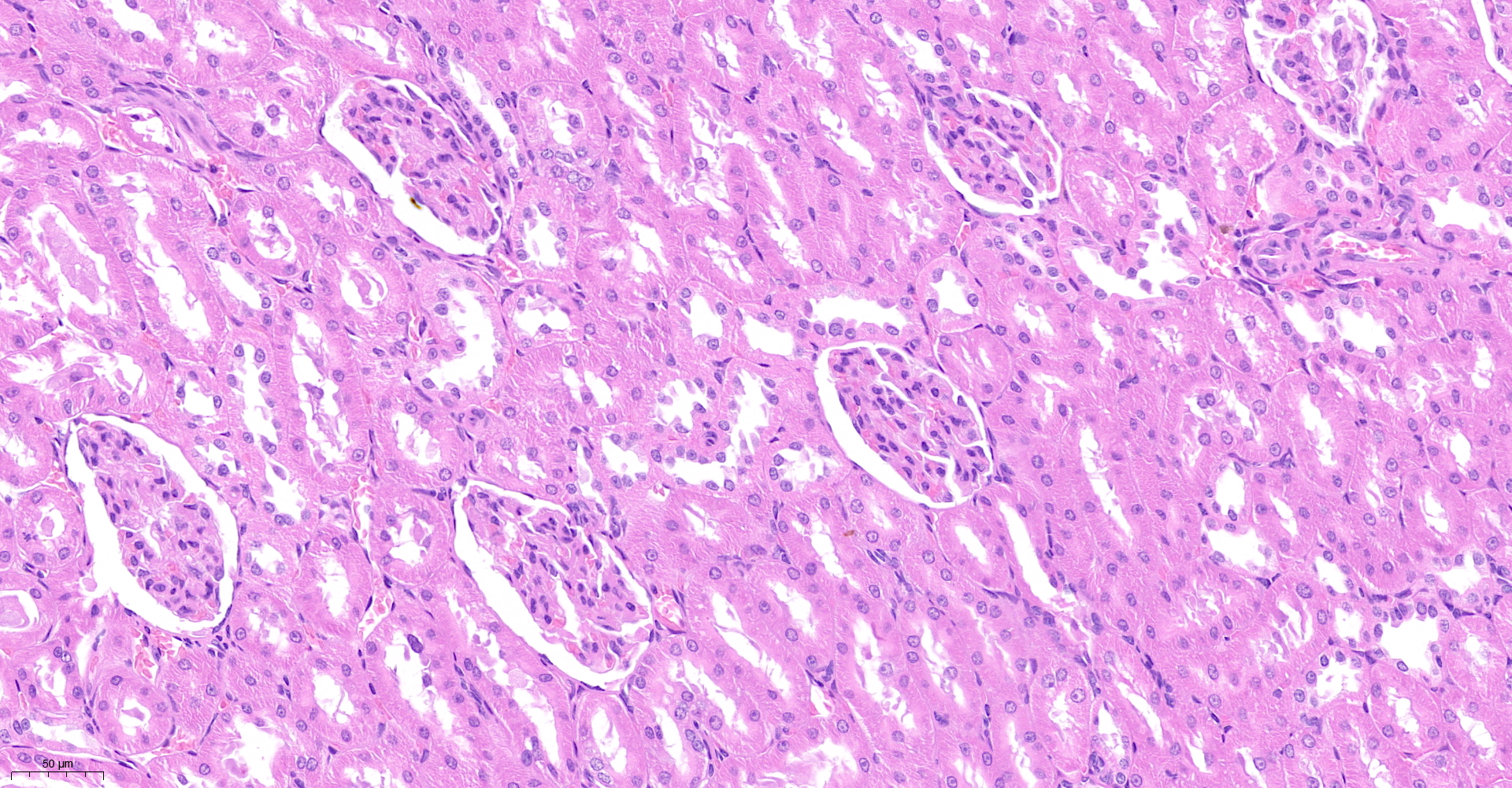


Cd 400×


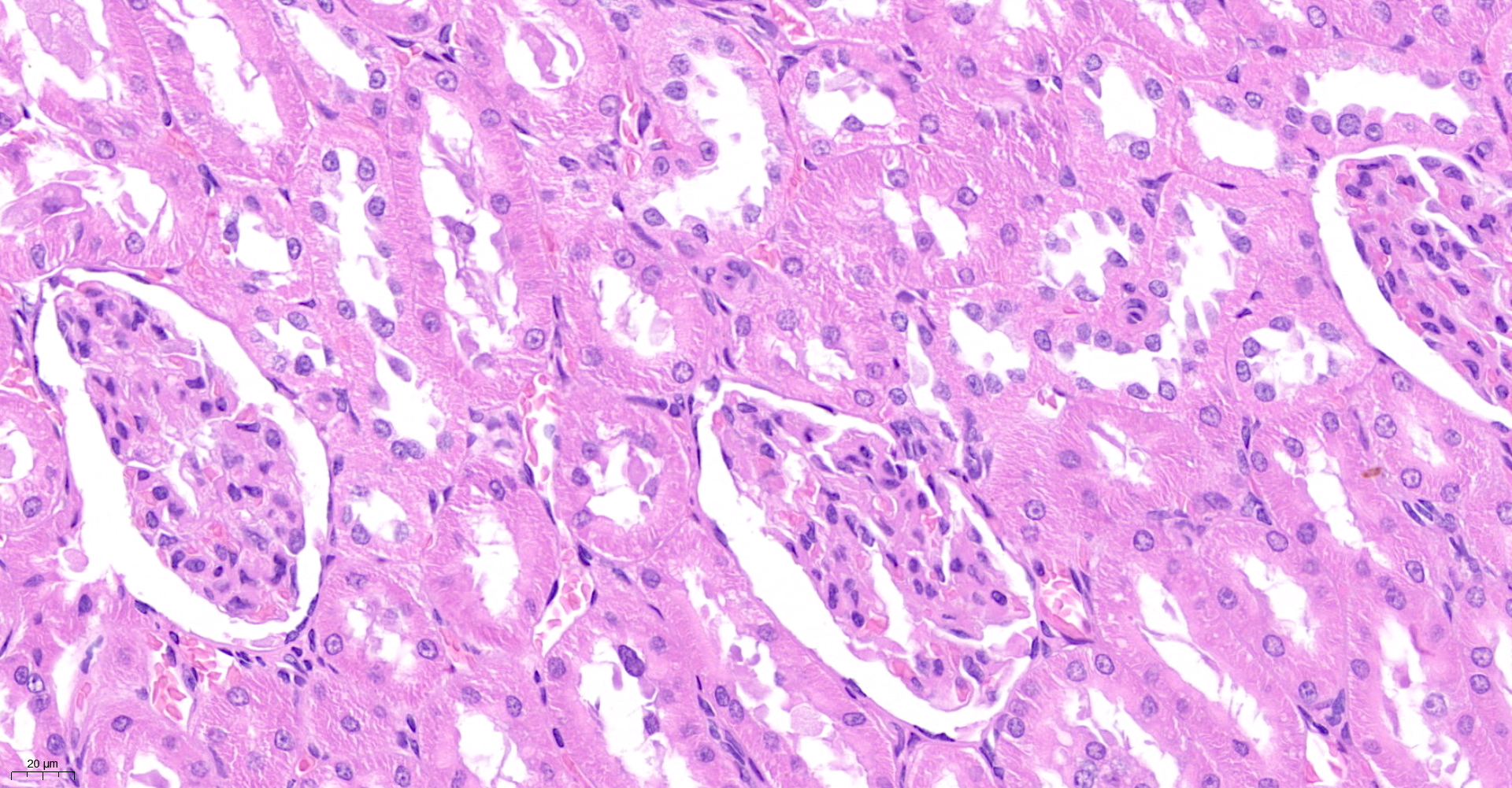


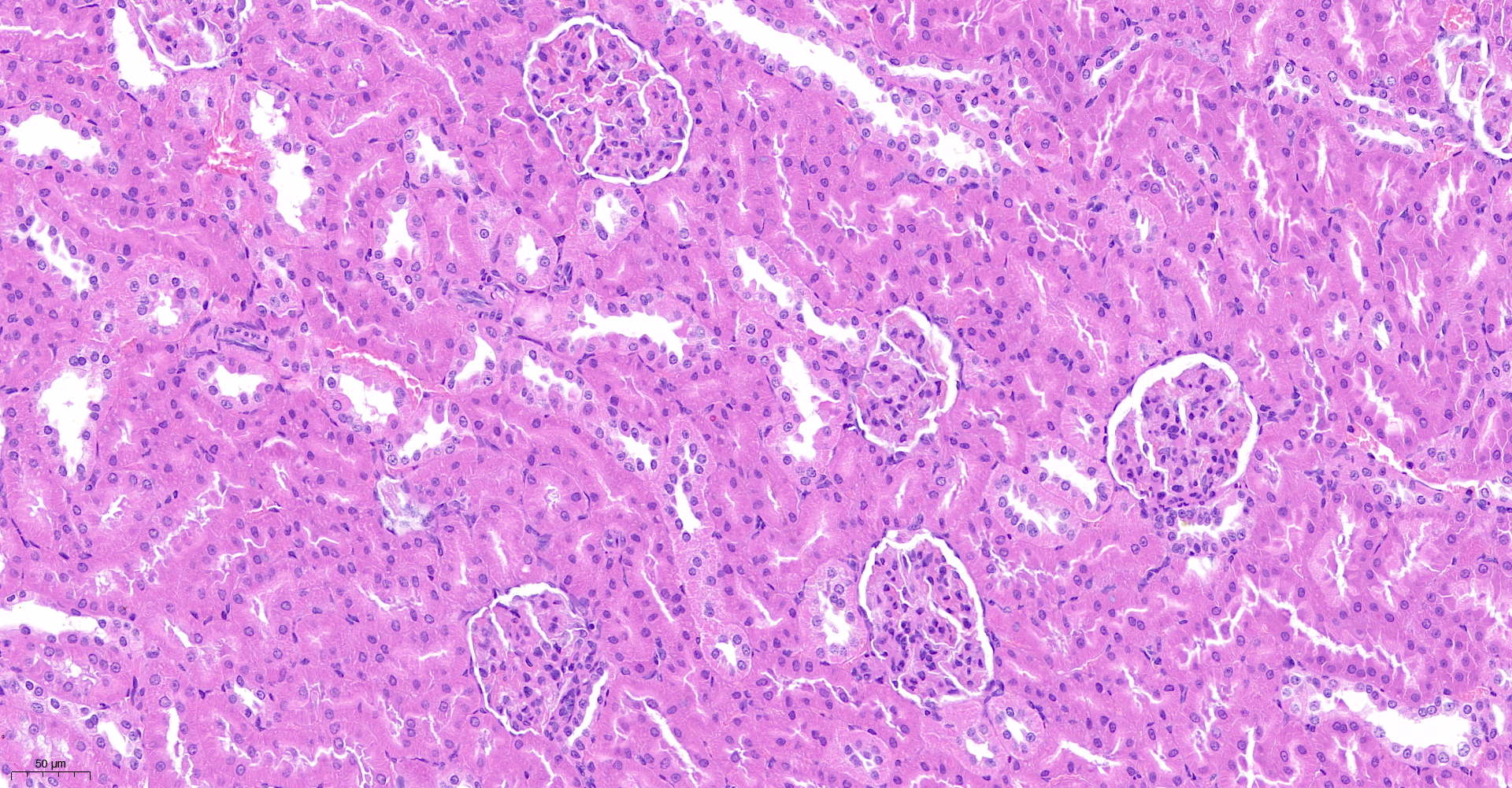
Cd + Que 200×


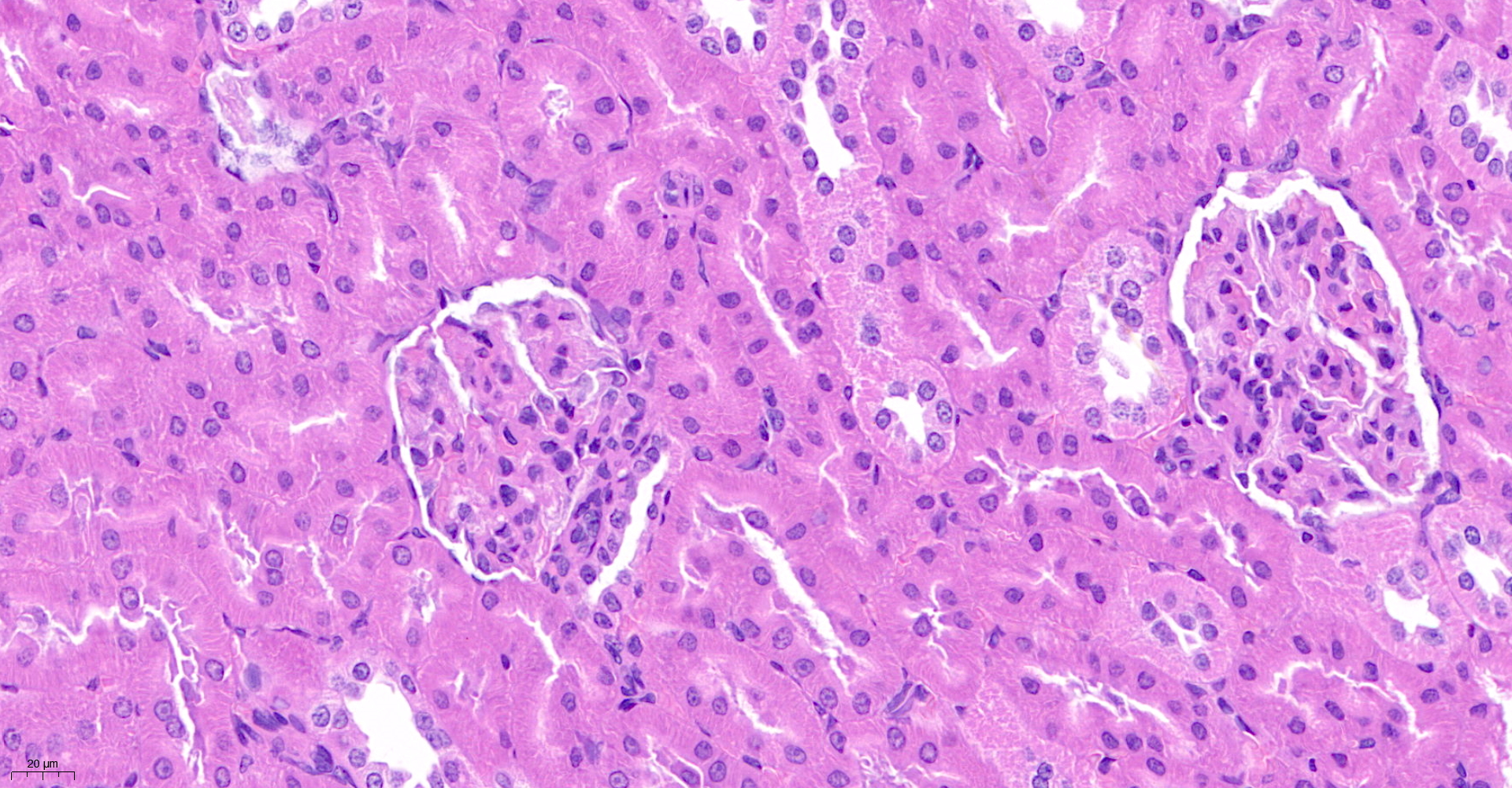


Cd + Que 400 ×

Que 200×


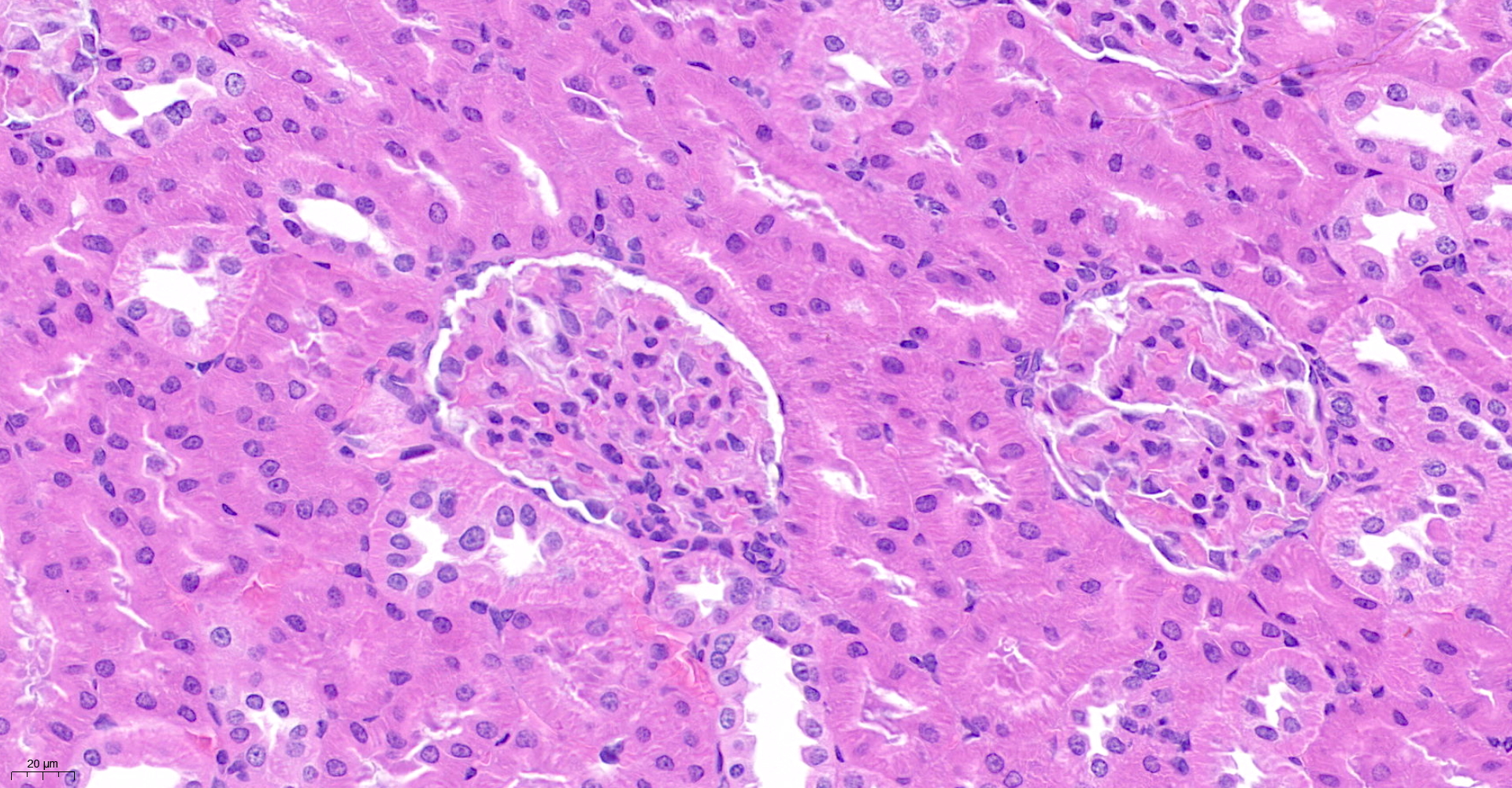


Que
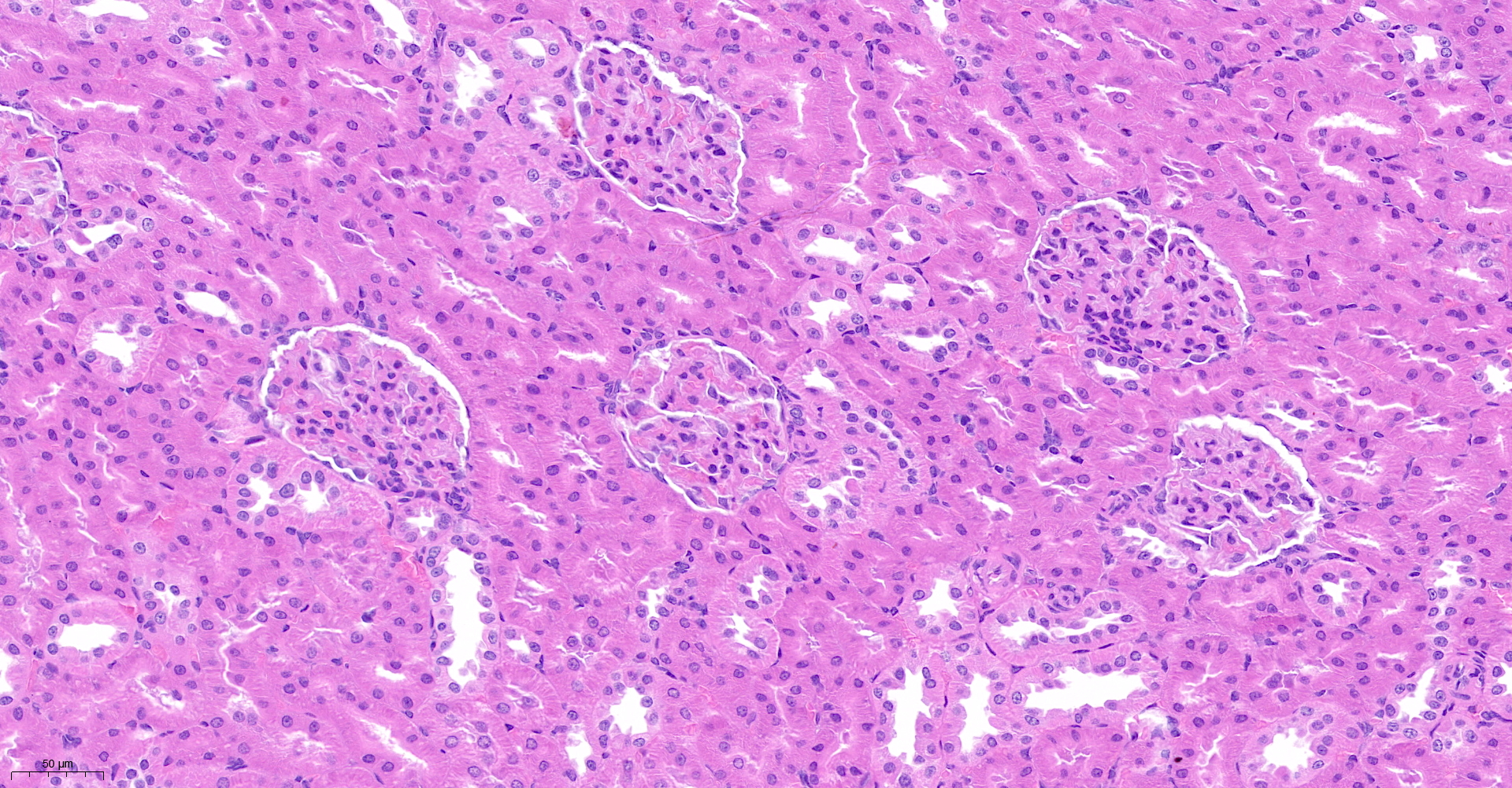
 400×

Supplement: Supplementary file 2 [file Table6.DOC]

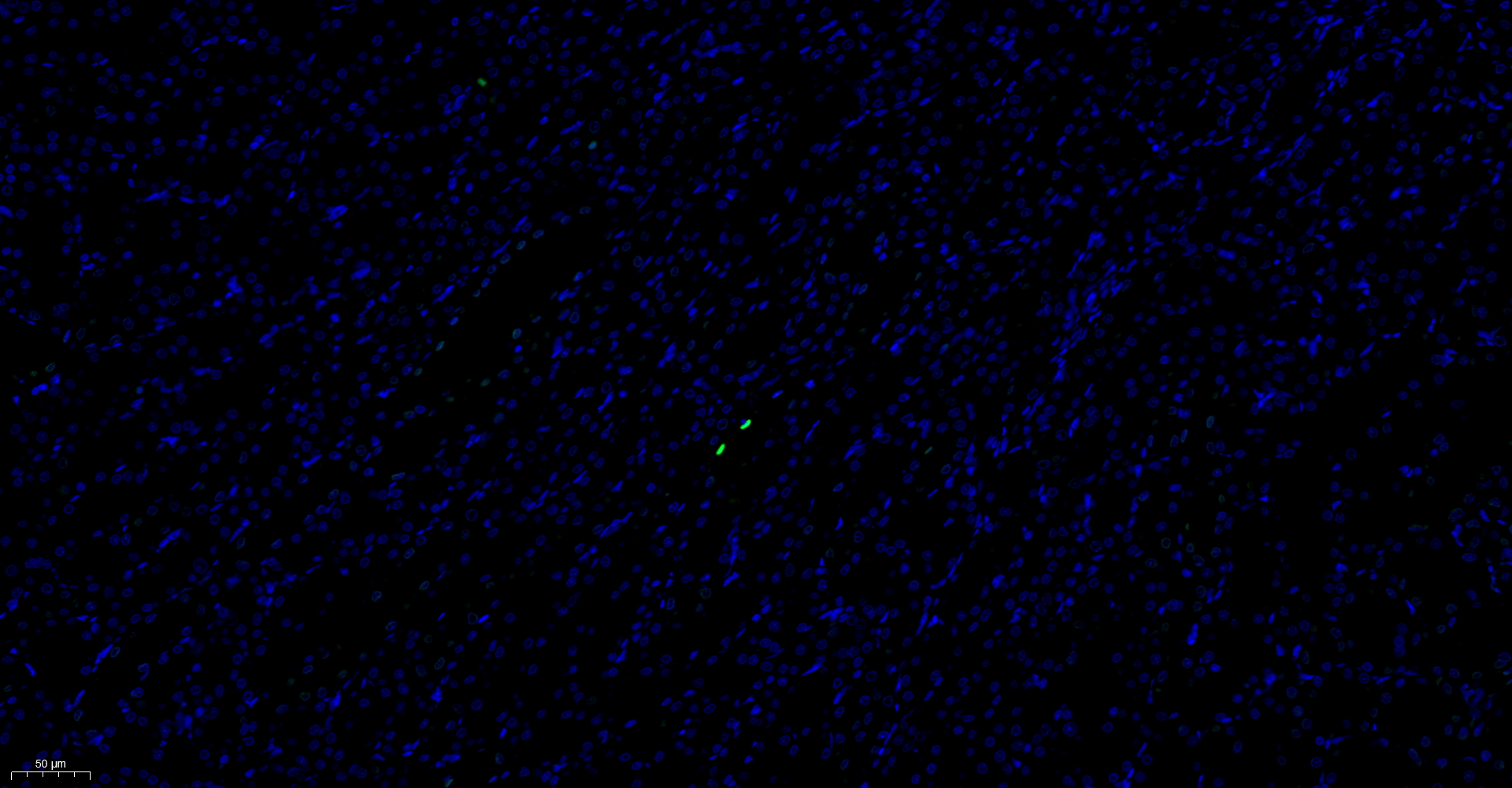

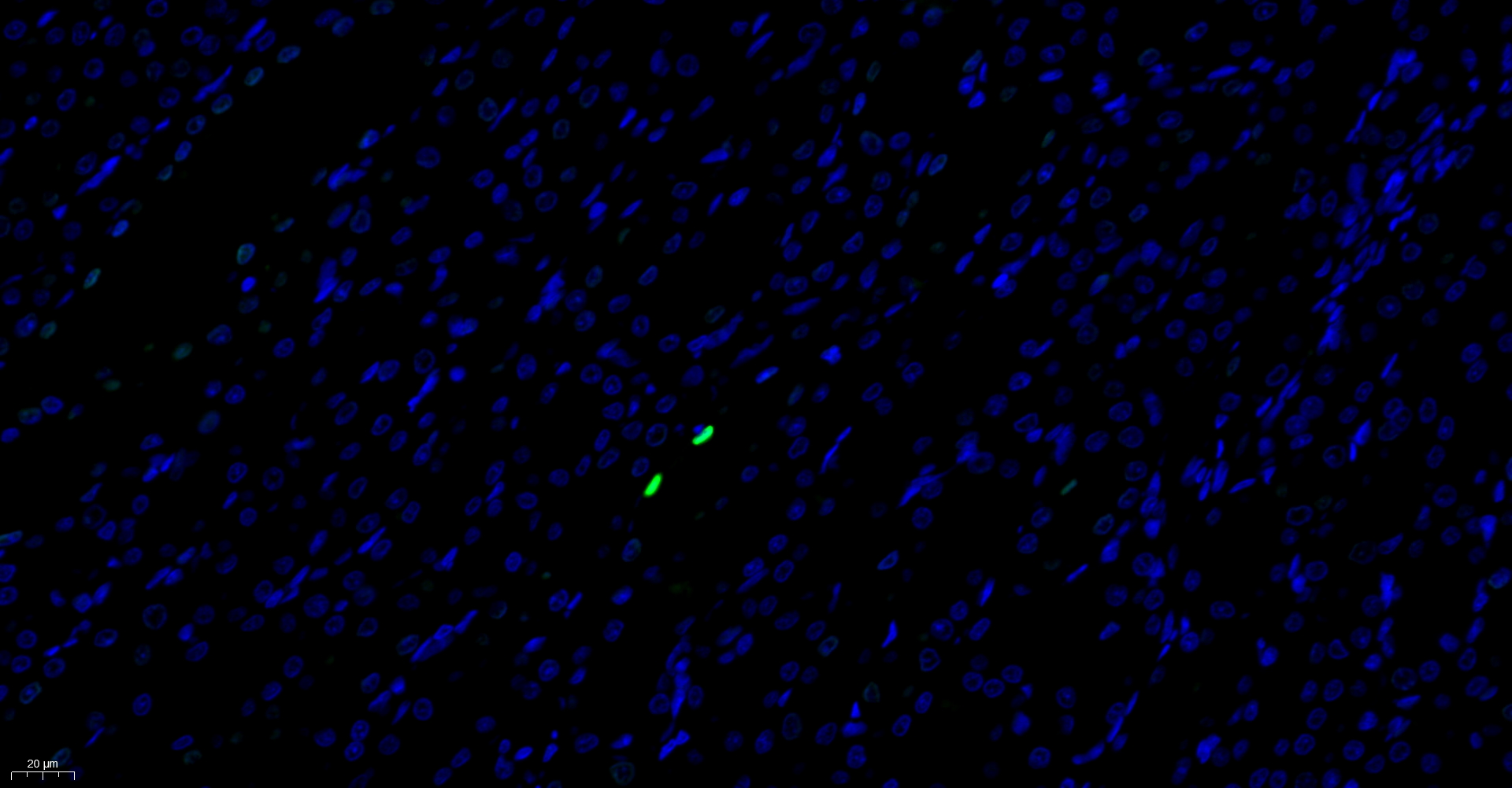
control 200×


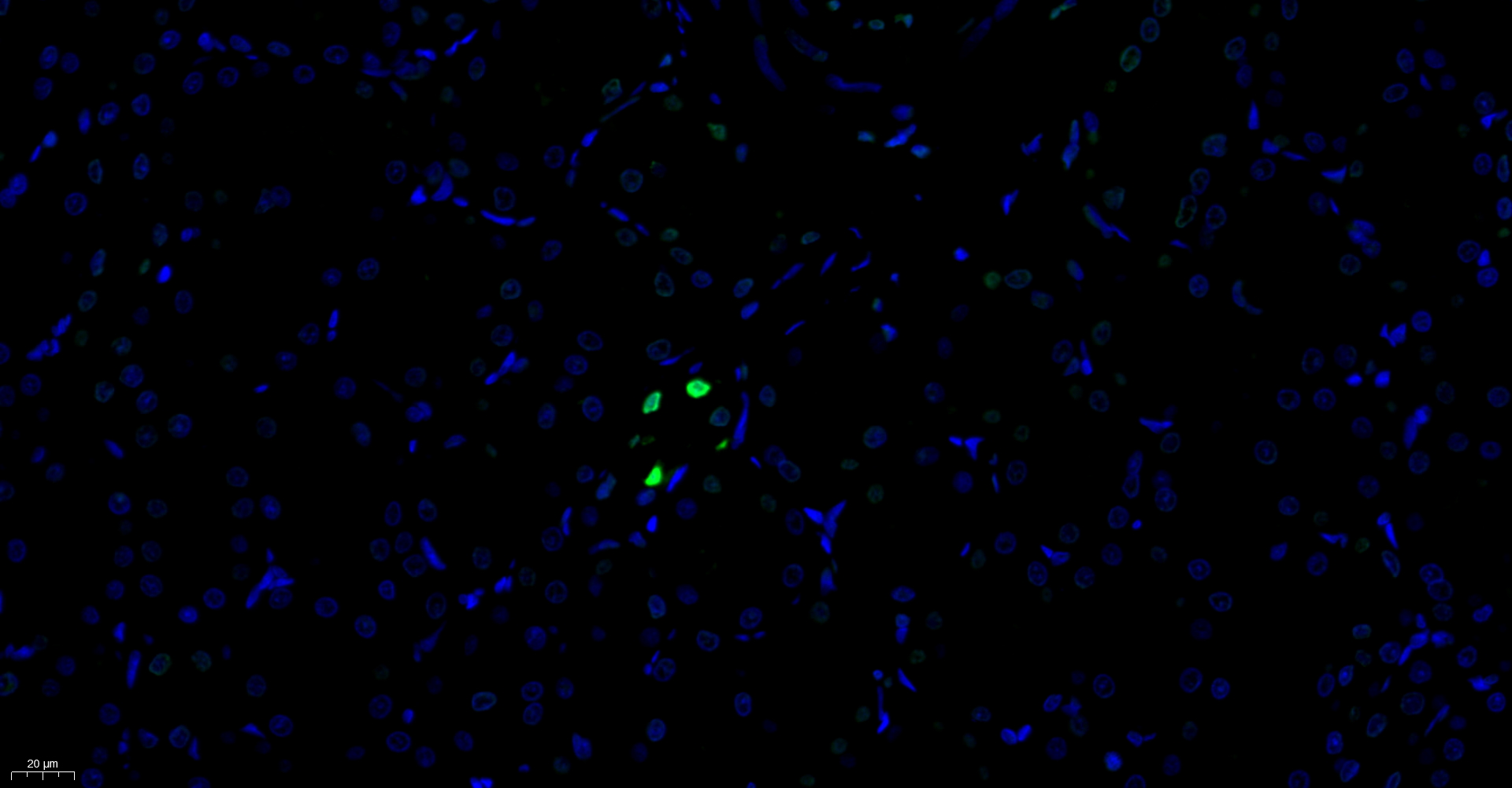
control 400× （1）

control 400× （2）

control 400× （3
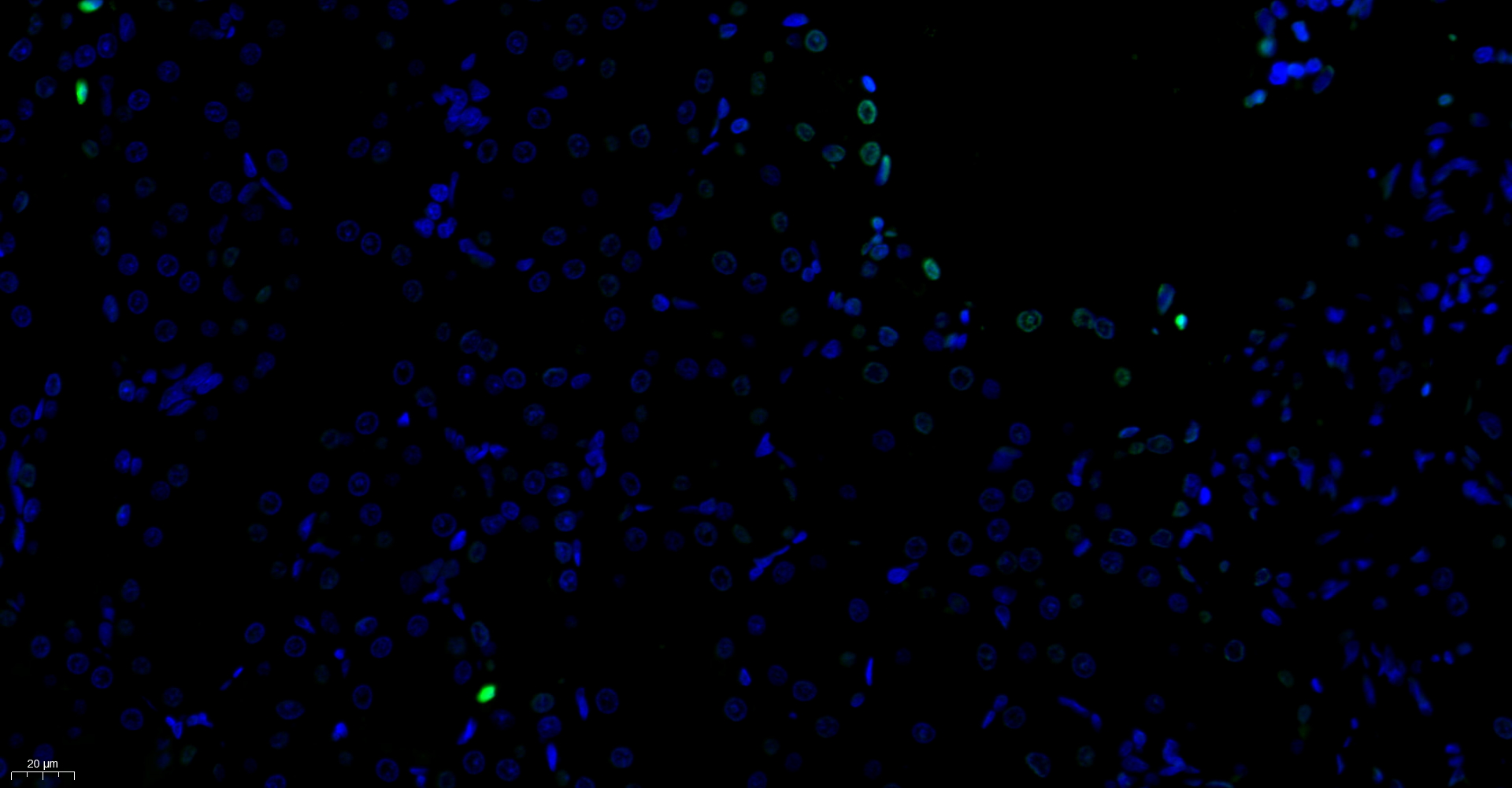
）


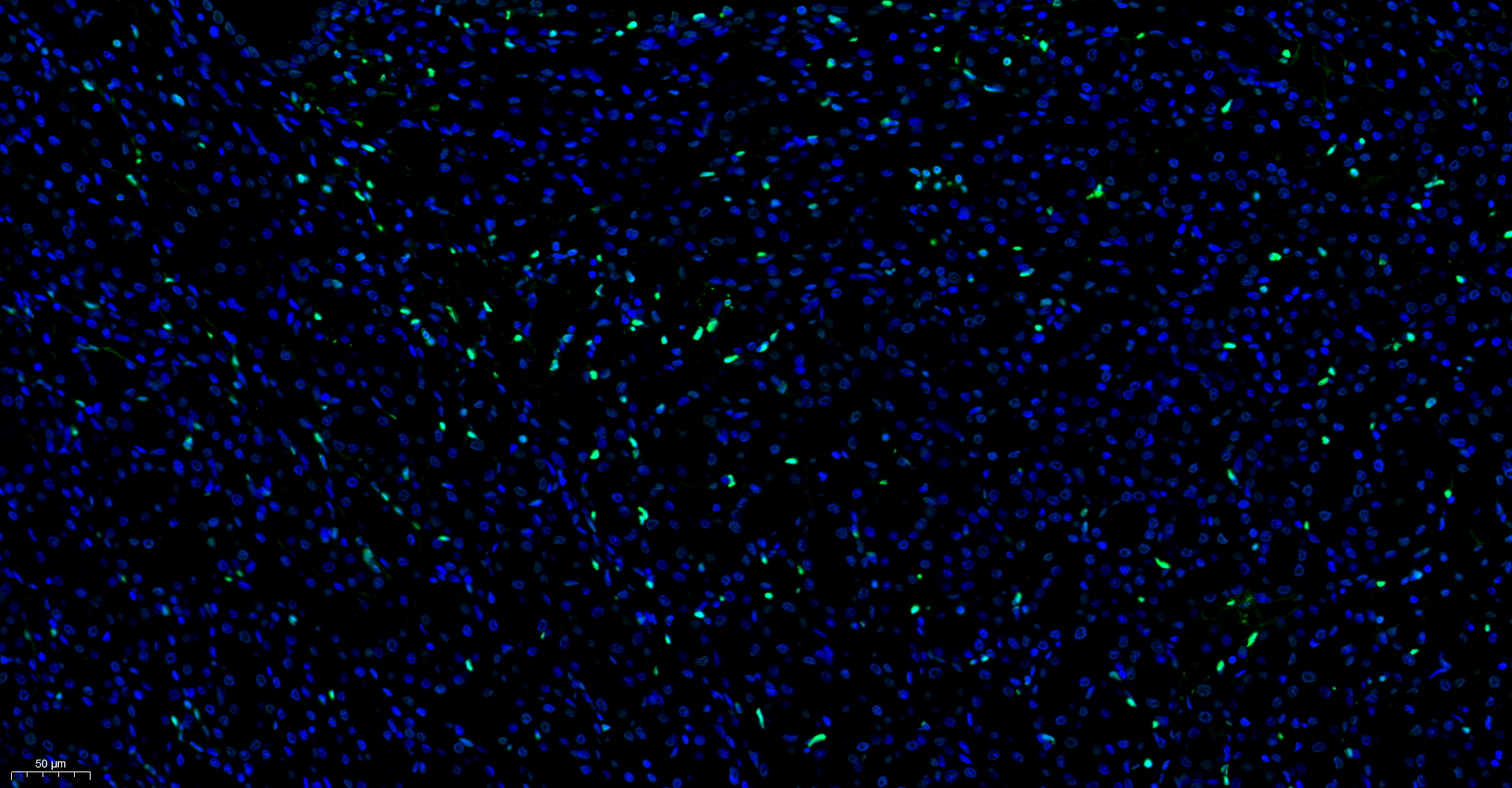

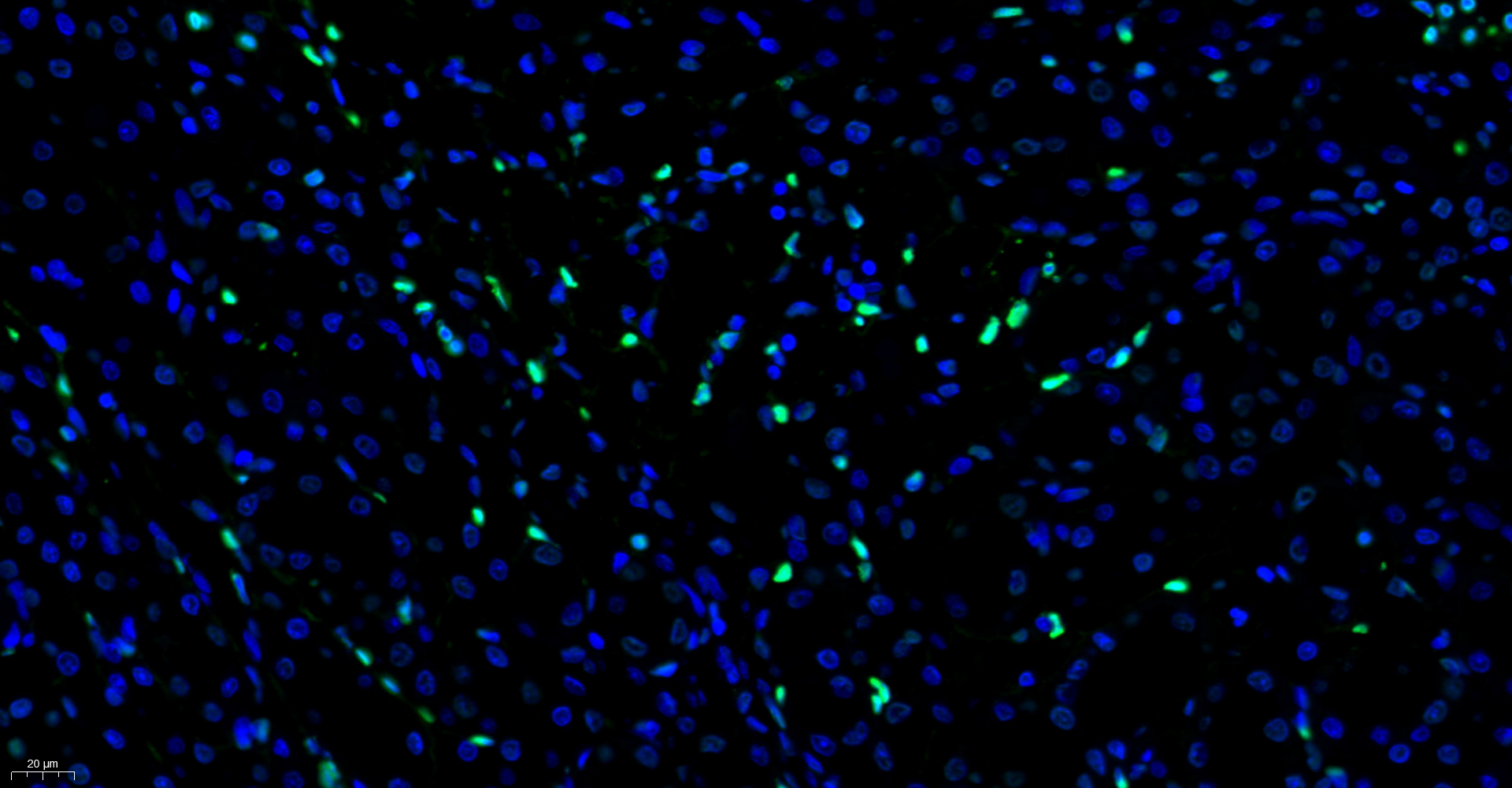
Cd 200×


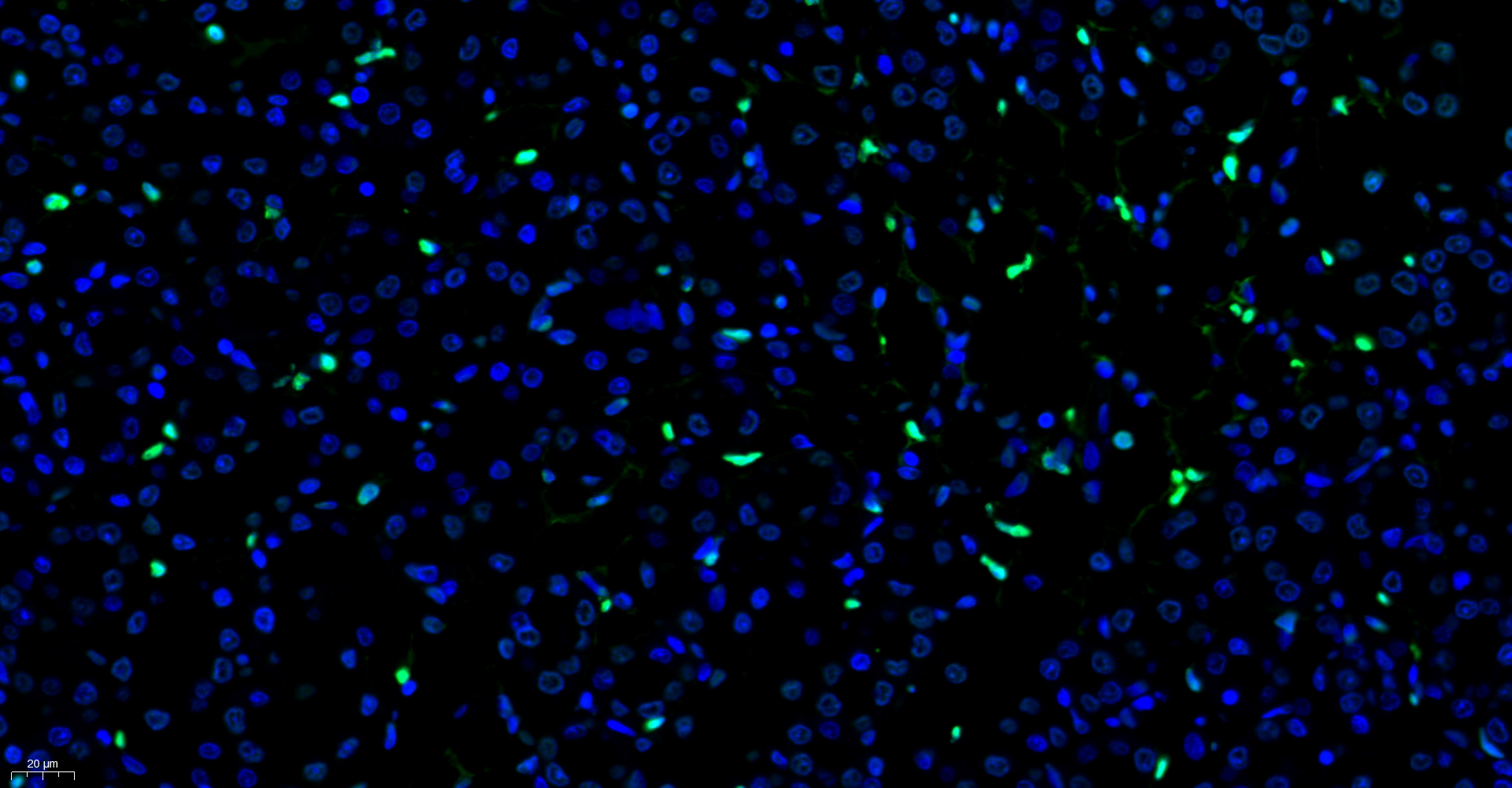
Cd 400× （1）


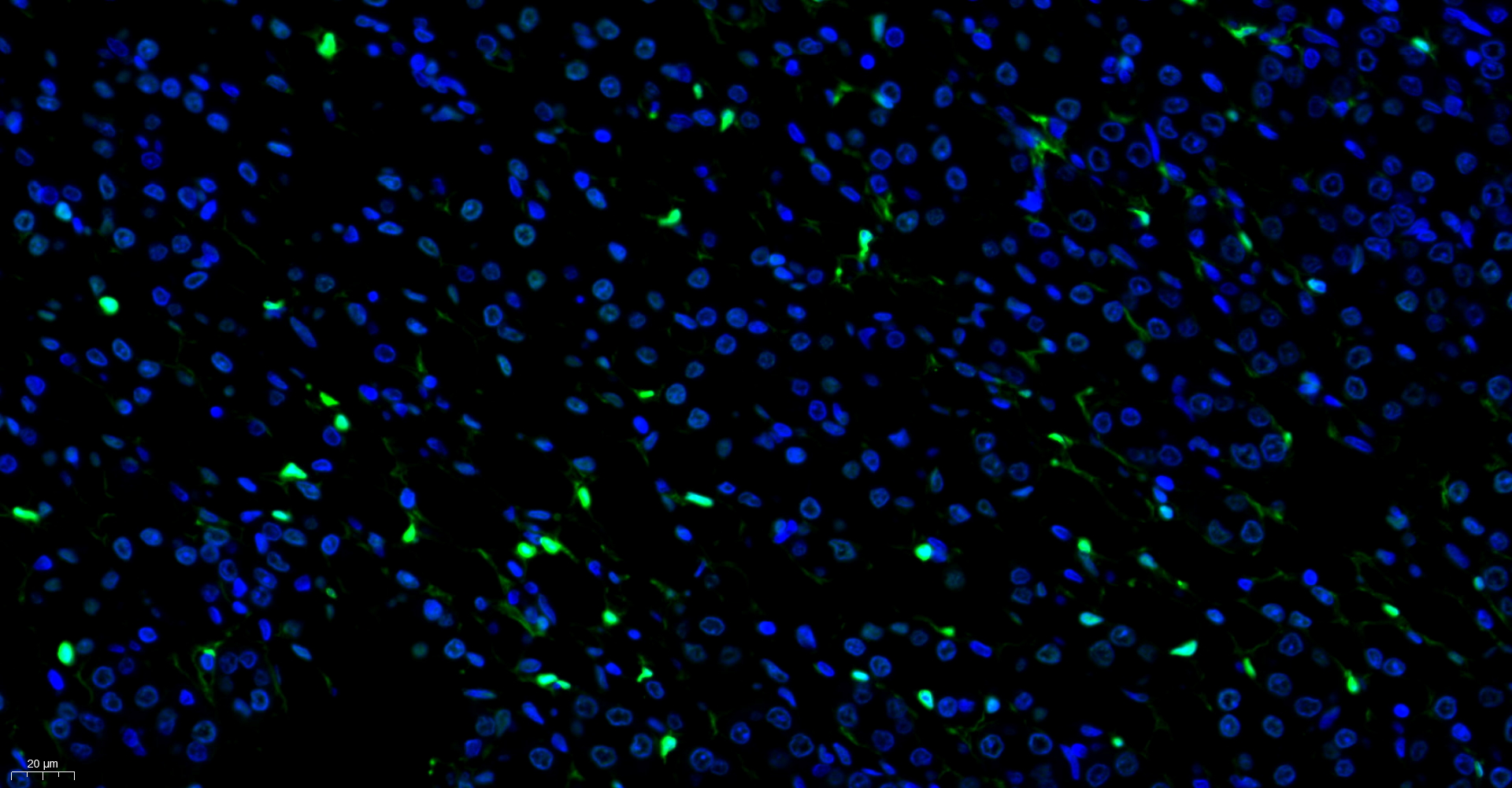
Cd 400× （2）

Cd 400× （3）


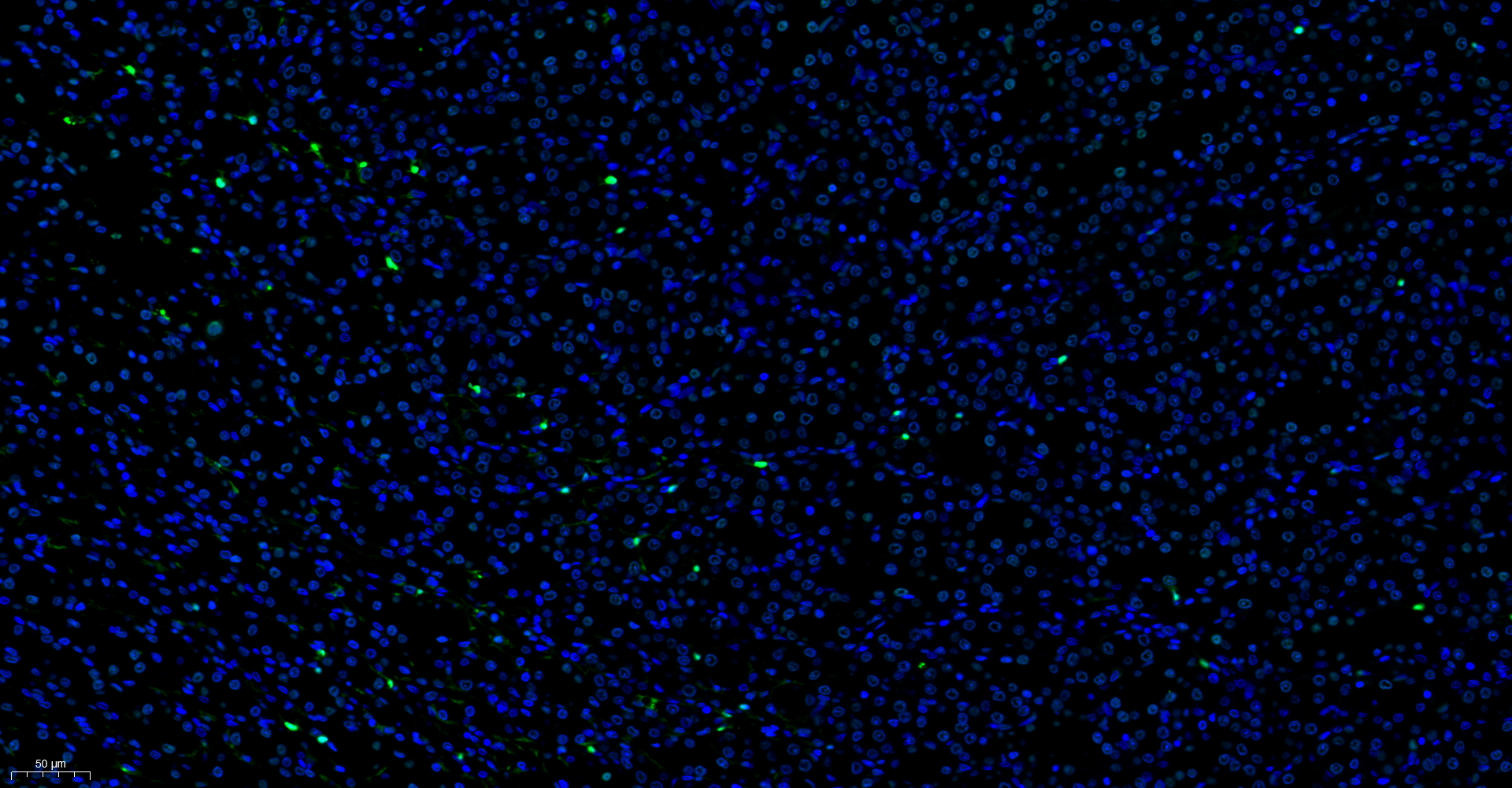

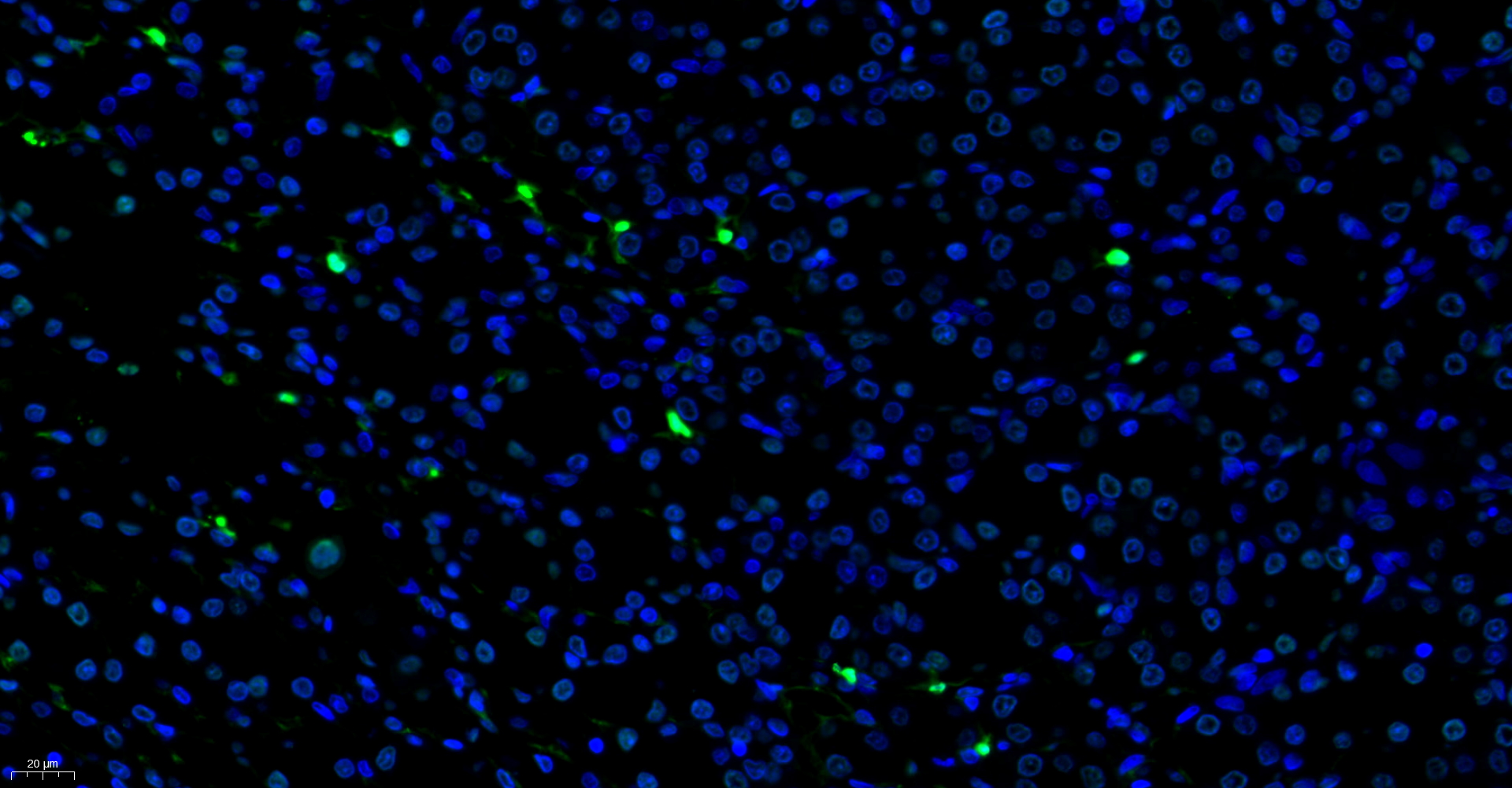
Cd + Que 200×


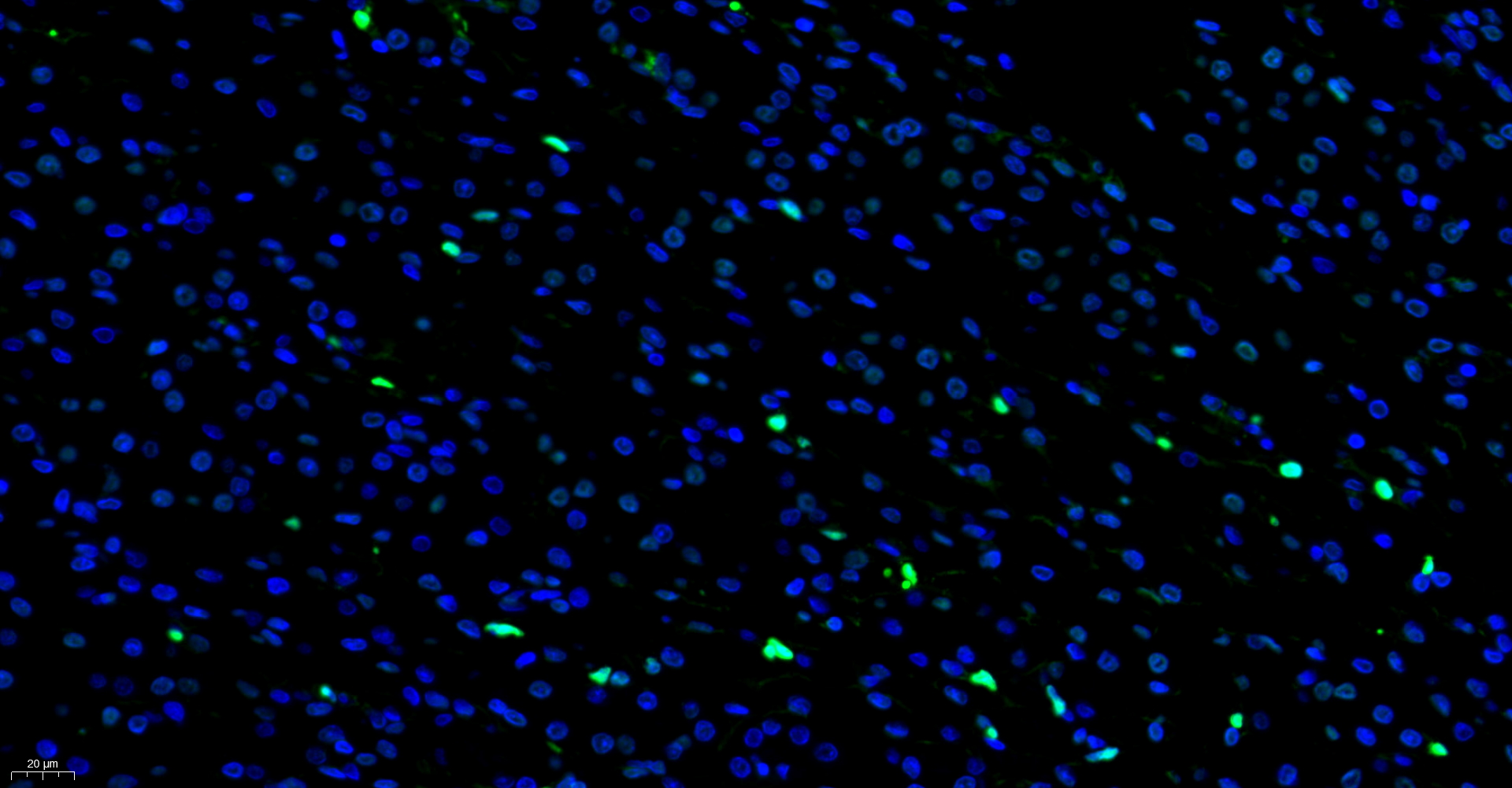
Cd + Que 400× （1）


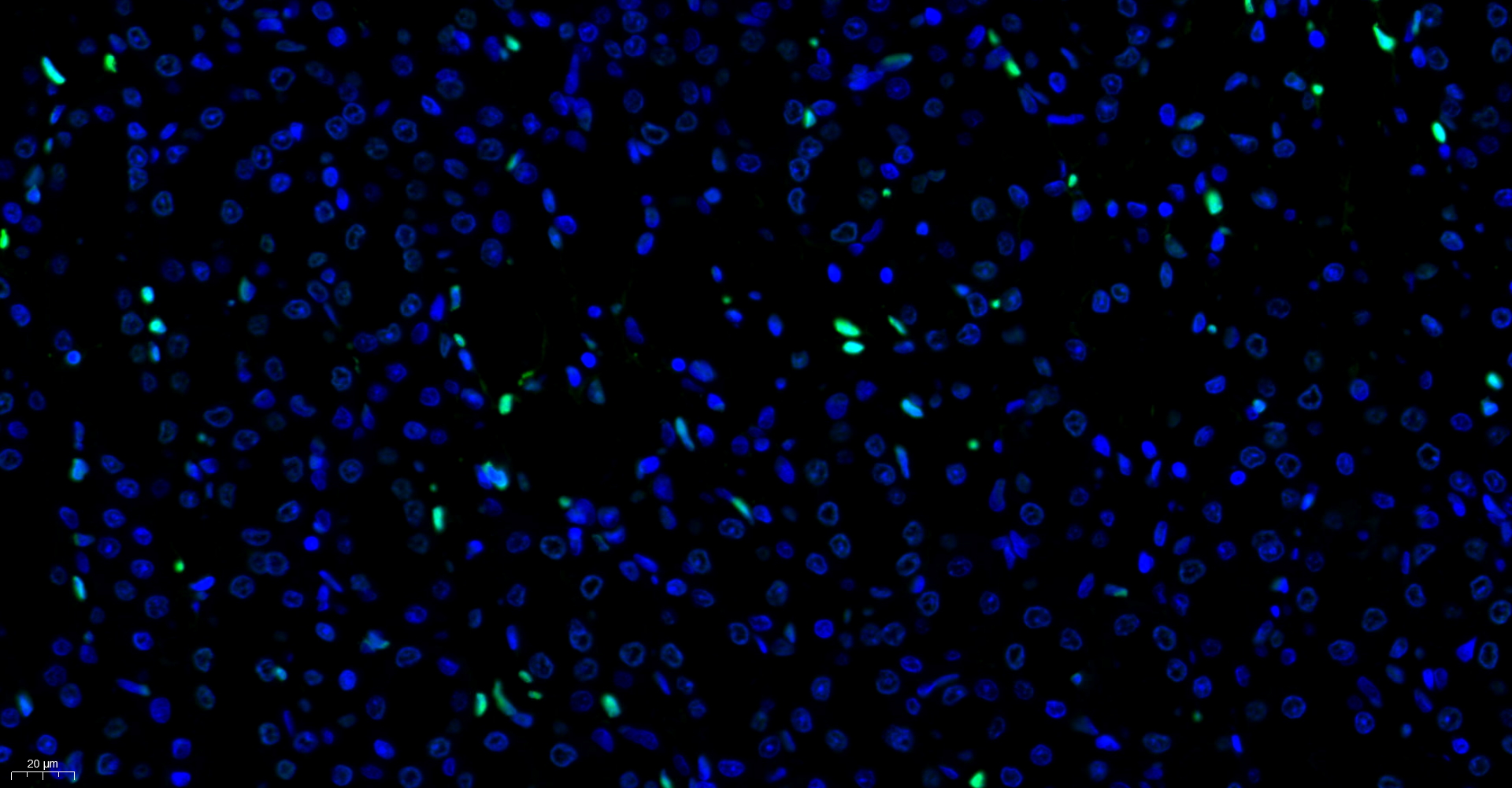
Cd + Que 400× （2）

Cd + Que 400× （3）


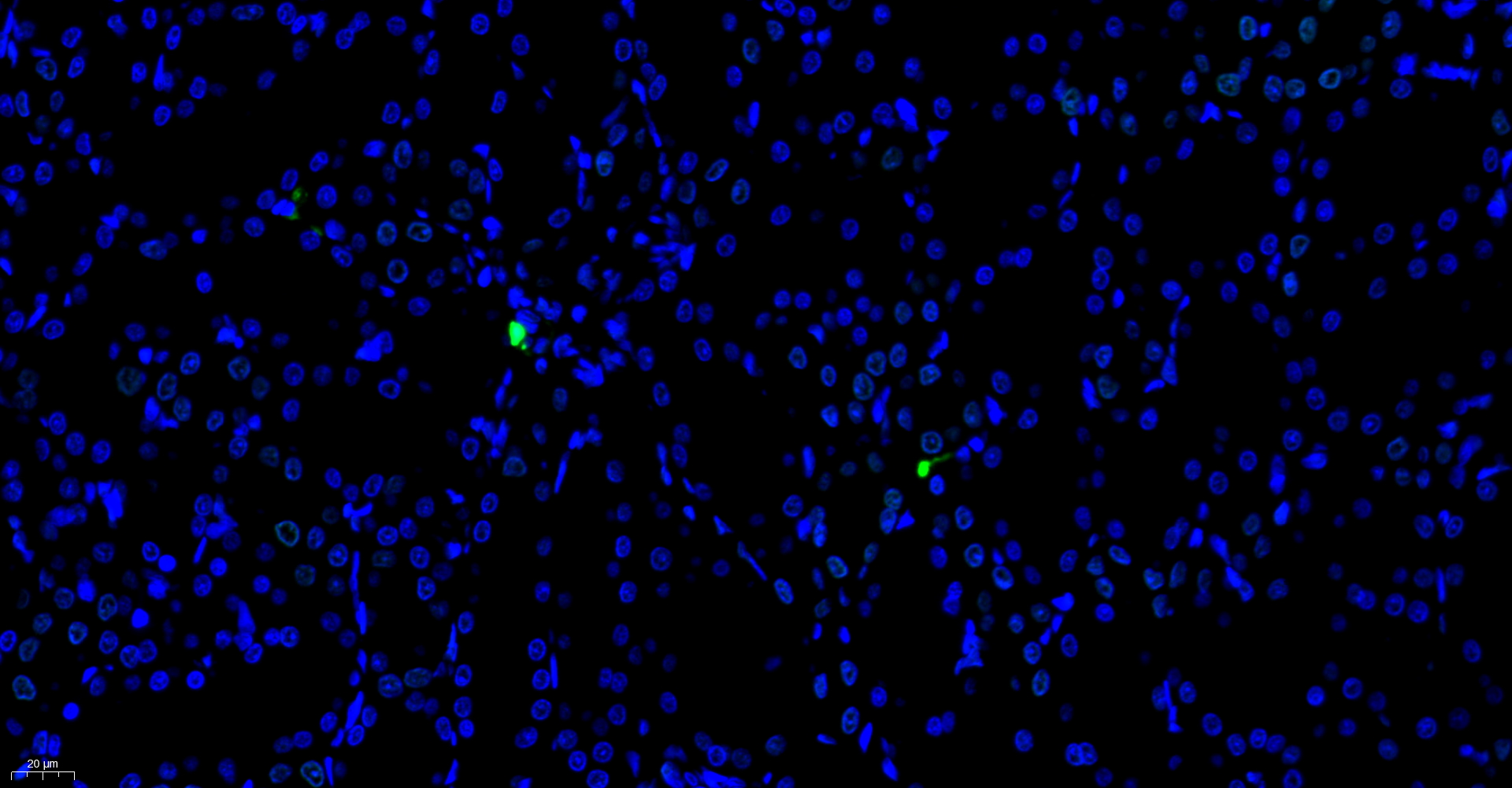
Que
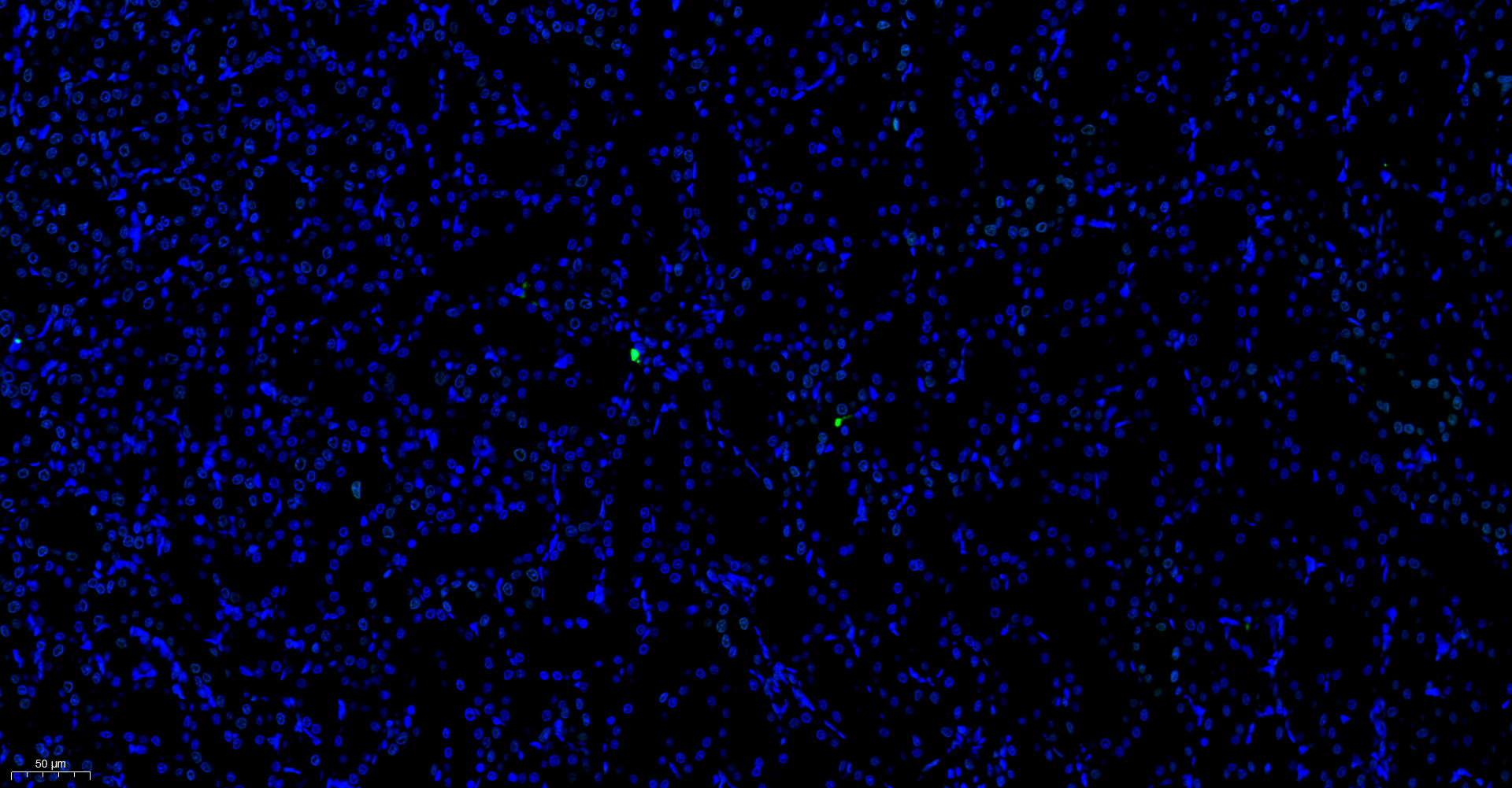
 200×


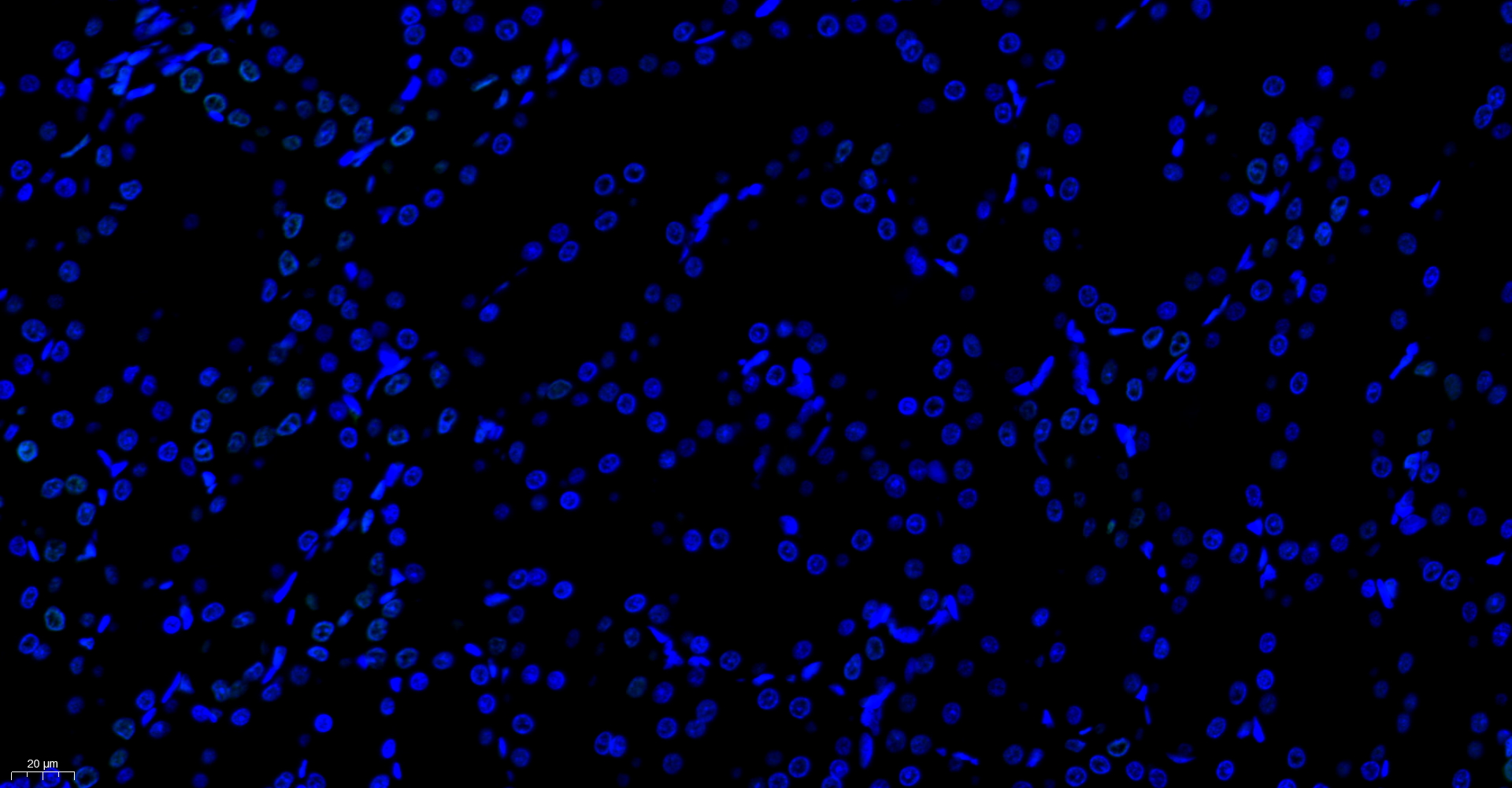
Que 400× （1）


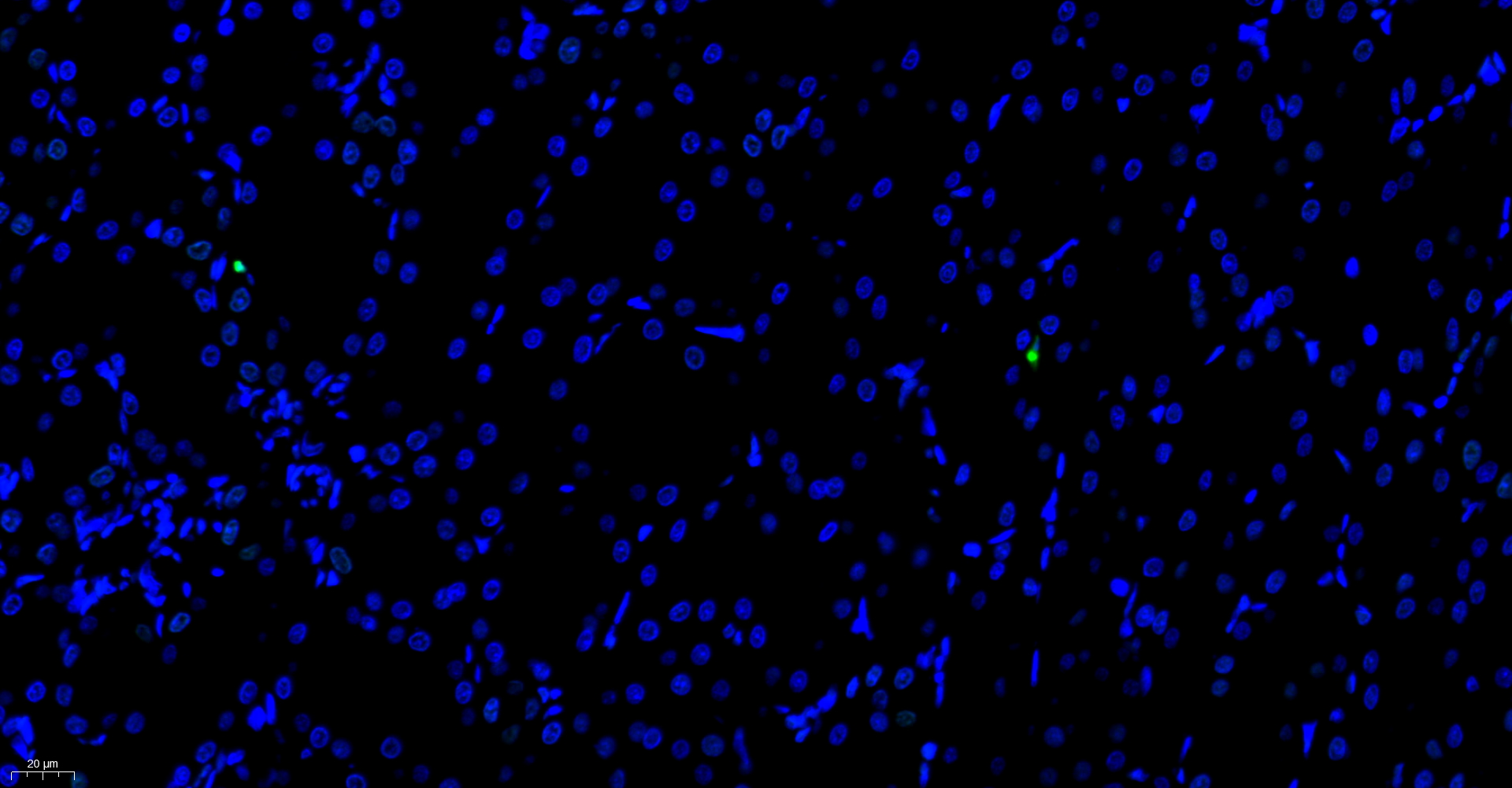
Que 400× （2）

Que 400× （3）

Supplement: Supplementary file 4 [file Table5.DOC]

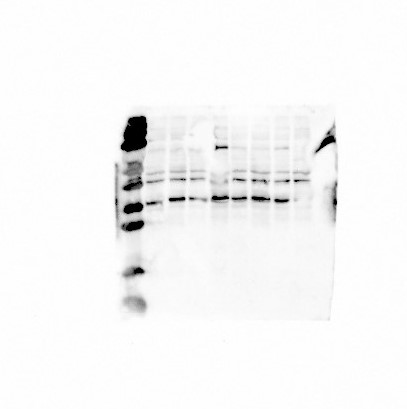


**Caspase 9**

Cd - + + -

Quercetin - - + +


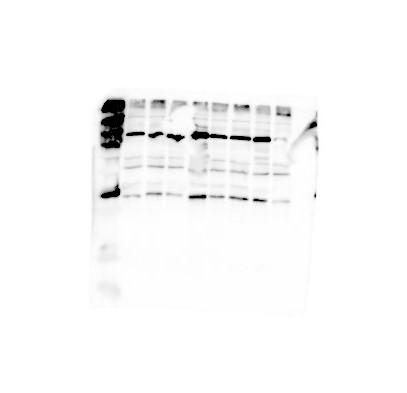


Cd - + + -

Quercetin - - + +

**Caspase 3**


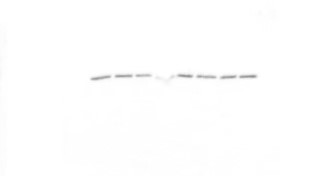


**β-Actin**

Cd - + + -

Quercetin - - + +


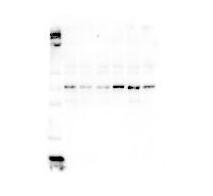


**Cyt-c**

Cd - + + -

Quercetin - - + +

Supplement: Supplementary file 6 [file Table4.DOCX]
